# Thermally-Induced Spin Crossover and LIESST Effect in the Neutral [Fe^II^(^Me^bik)_2_(NCX)_2_] Complexes: Variable-Temperature Structural, Magnetic, and Optical Studies (X = S, Se; ^Me^bik = bis(1-methylimidazol-2-yl)ketone)

**DOI:** 10.3389/fchem.2018.00326

**Published:** 2018-08-21

**Authors:** Siddhartha De, Lise-Marie Chamoreau, Hasnaa El Said, Yanling Li, Alexandrine Flambard, Marie-Laure Boillot, Subrata Tewary, Gopalan Rajaraman, Rodrigue Lescouëzec

**Affiliations:** ^1^Sorbonne Université, CNRS, Institut Parisien de Chimie Moléculaire, Paris, France; ^2^Institut de Chimie Moléculaire et des Matériaux d'Orsay, CNRS, Bat 420, Univ. Paris-Sud, Université Paris-Saclay, Orsay, France; ^3^Department of Chemistry, Indian Institute of Technology Bombay, Mumbai, India

**Keywords:** spin crossover, iron(II) complexes, photomagnetism, DFT, B3LYP/B3LYP^*^

## Abstract

Two new iron(II) neutral complexes of bis(1-methylimidazol-2-yl)ketone (^Me^bik) with molecular formula [Fe^II^(^Me^bik)_2_(NCS)_2_] (**1**) and [Fe^II^(^Me^bik)_2_(NCSe)_2_] (**2**) have been synthesized and characterized by magnetic measurements, single-crystal X-ray diffraction, and solid state UV-vis spectroscopy. The temperature dependent magnetic susceptibility measurements of crystalline samples of both compound show the occurrence of a gradual spin transition centered at *T*_1__/2_ = 260 K and 326 K, respectively. The crystal structures of both compounds were determined at different temperatures, below and above the transition, in order to detect the structural changes associated with the spin transition. The main structural modifications, when passing from the low-spin to the high-spin form, consist of an important lengthening of the Fe-N(Mebik) and Fe-N (C-S/Se) distances (by ca. 0.20 and 0.18 Å, respectively) and a noticeable variation of the N-Fe-N angles, leading to a more distorted [Fe-N6] octahedron. The spin-transition phenomenon also affects the optical properties, with significant decrease of the intensity of the Metal-to-Ligand charge transfer band upon increasing the temperature. Finally, both complexes exhibit a light-induced excited spin-state trapping under laser light irradiation at low temperature. DFT calculations were also carried out on these complexes in order to rationalize the theoretically predicted magnetic and optical behavior with those of the experimental one. The results clearly highlights the dramatic alteration of the magneto-structural behavior of the tris-chelate ${\left[{\text{Fe}}^{\text{II}}{{(}^{\text{Me}}\text{bik})}_{3}\right]}_{2}^{+}$ spin-crossover complex upon substituting one Mebik with NCS and NCSe ligands.

## Introduction

The rational design of switchable molecular materials featuring stimuli-responsiveness is attracting strong research efforts because of the potential use as molecular sensors, switches, actuators, or memories in future molecular devices (Sauvage and Amendola, 2001; Feringa, 2011; Shepherd et al., 2013; Ferrando-Soria et al., 2017). The spin-crossover (SCO) complexes represent an emblematic class of switchable molecular systems. In these complexes, the application of thermal variation, pressure, light-irradiation, electric, or magnetic fields can induce an electronic change between a low-spin (LS) and a high-spin (HS) state, which leads to important changes in both optical and magnetic properties (Halcrow, 2013). The Fe(II) SCO complexes are of particular interest as the electronic reorganization involves a transition between a diamagnetic low-spin state (S = 0, ${\text{t}}_{2\text{g}}^{6}$) and a paramagnetic high-spin state (S = 2, ${\text{t}}_{2\text{g}}^{4}$${\text{e}}_{\text{g}}^{2}$). The spin-state change is also accompanied by a structural reorganization, in particular with a significant lengthening of the Fe-N(ligand) distances (*ca*. 0.2 Å). The important structural reorganization have been shown to favor the Light-Induced Spin-State Trapping (LIESST) and photomagnetic effects are often observed in Fe(II) SCO complexes (Létard, 2006).

**Scheme 1:**
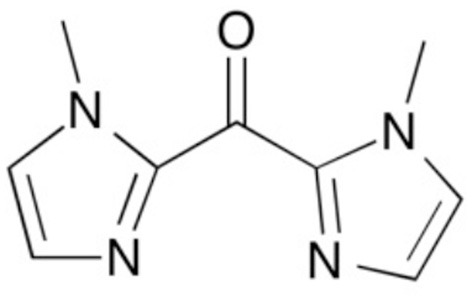
bis(1-methylimidazol-2-yl)ketone, ^Me^bik.

In recent years, we have been interested by the use of the bis(1-R-imidazol-2-yl)ketone ligands (with R = methyl, ethyl, vinyl), named “^R^bik,” for designing switchable photomagnetic molecules (Scheme 1). From a structural point of view, the ^R^bik ligands are β di-imine, which can be compared to the well-known α di-imine ligands: L-L = 2,2'-bipyridine (bipy) and 1,10-phenanthroline (phen). The presence of the ketone group between the two imidazolyl donor groups confers some aromaticity to these ligands, which are thus π-acceptor ligands, like the bipy and phen. However ^R^bik ligands form six-membered chelate rings leading to weaker ligand field than for the five membered chelate rings observed in similar bipy and phen complexes. Thus, whereas [Fe^II^(L-L)_3_]^2+^ are a low-spin complexes, the [Fe^II^(^R^bik)_3_]^2+^ are SCO complexes (De et al., 2018). The high-spin six-membered rings [Fe^II^(^R^bik)_3_]^2+^ complexes are also more labile at room temperature in comparison to the inert [Fe^II^(L-L)_3_]^2+^, which makes the preparation of bis-chelate complexes more easy. Taking advantage of such feature, we synthesized and studied [Fe(^R^bik)_2_(NC-)_2_] SCO systems, where one of the ^R^bik ligand is replaced by two cyanido metallo-ligand, abbreviated (NC-). For example, we investigated two tetranuclear rhombus {Fe_2_M_2_} (M = Fe, Mo) switchable complexes containing the {Fe^II^(^R^bik)_2_(NC-)_2_} subunit (NC- represents cyanido metallo ligand: [Fe^III^(Tp)(CN)_3_]^−^ or [Mo^V^(CN)_8_]^3−^) (Mondal et al., 2012, 2014). Both complexes show gradual thermally-induced spin transition on the Fe(II) centers and photomagnetic effect (LIESST) at low temperature. In the {[Fe^III^(Tp)(CN)_3_]_2_[Fe^II^(^Me^bik) _2_]_2_} [Fe^III^(Tp)(CN)_3_]_2_·18H_2_O·4CH_3_OH compound, the transition is centered at *ca. T*_1__/2_ = 330 K whereas in the {[Mo(CN)_8_]_2_[Fe(^Me^bik)_2_]_2_}(^H^MeIm)_2_·5H_2_O·CH_3_CN compound, it occurs at a slightly higher temperature, *ca. T*_1__/2_ = 350 K. In both cases the transition is significantly shifted toward lower temperature (*ca*. 250 K for the two compounds) and becomes more gradual upon solvent removal. A striking difference between the two {Fe_2_M_2_} rhombus complexes and the [Fe^II^(^Me^bik)_3_](BF_4_)_2_ monometallic complex arises from the photomagnetic behavior, which was investigated using different laser diode in the visible and near IR range (404, 532, 605, 750, 808, 900 nm). Whereas the {Fe_2_Fe_2_} compound show a significant LIESST effect in the 700–900 nm range (only in the dehydrated phase), the {Fe_2_Mo_2_} shows the strongest increase of magnetization at 405 nm, whereas LIESST effect is much less significant at 800 nm. In contrast, the [Fe(^Me^bik)_3_](BF_4_)_2_ monometallic complex shows a maximal effect at 635 nm (De et al., 2018).

In order to further extend these seminal investigation, the present contribution focuses on the switchable properties of the mononuclear SCO complexes [Fe^II^(^Me^bik)_2_(NCX)_2_] (X = S, Se), which can be considered as model compounds for our previously reported systems as the group NC- is here a simple inorganic ligand. We thus present here a structural, spectroscopic, (photo)magnetic study of these two new spin crossover compounds: [Fe^II^(^Me^bik)_2_(NCS)_2_] (1) and [Fe^II^(^Me^bik)_2_(NCSe)_2_] (2).

## Results and discussion

### Synthesis

Both compounds were prepared similarly, by simply reacting *in-situ* prepared [Fe(^Me^bik)_2_(S)_2_] complex (S = solvent) with a stoichiometric amount of NCX salts in air and at room temperature (see details in the experimental part).

### Description of the crystal structure

The single crystal X-ray diffraction structure of **1** and **2** were determined at 200(2), 300(2), and 400(2) K. The compounds are isostructural and crystallize in the monoclinic C2/c space group (Z = 4). Selected crystallographic data are reported in Table 1 and Tables S1, S2.

**Table 1:** Cell parameters and volume cells of 1 and 2 obtained at 100, 200 and 400 K.

	**[Fe(**^**Me**^**bik)**_**2**_**(NCS)**_**2**_**]**			**[Fe(**^**Me**^**bik)**_**2**_**(NCSe)**_**2**_**]**		
T / [K]	200 (2)	300 (2)	400 (2)	200 (2)	300 (2)	400 (2)
*a* [Å]	7.7745 (2)	7.6476 (2)	7.6806 (3)	7.9156 (10)	7.9181 (10)	7.8489 (4)
*b* [Å]	13.7943 (3)	14.4341 (4)	14.5138 (6)	13.8415 (2)	13.9977 (2)	14.5090 (6)
*c* [Å]	22.3611 (5)	22.3945 (6)	22.5939 (10)	22.5668 (4)	22.6609 (4)	22.9089 (9)
β [°]	100.564 (1)	99.280 (2)	99.281 (2)	99.7010 (10)	99.333 (10)	98.819 (3)
Volume [Å^3^]	2357.45 (10)	2439.69 (11)	2485.68 (18)	2437.15 (6)	2478.38 (6)	2578.0 (2)

**Figure 1:**
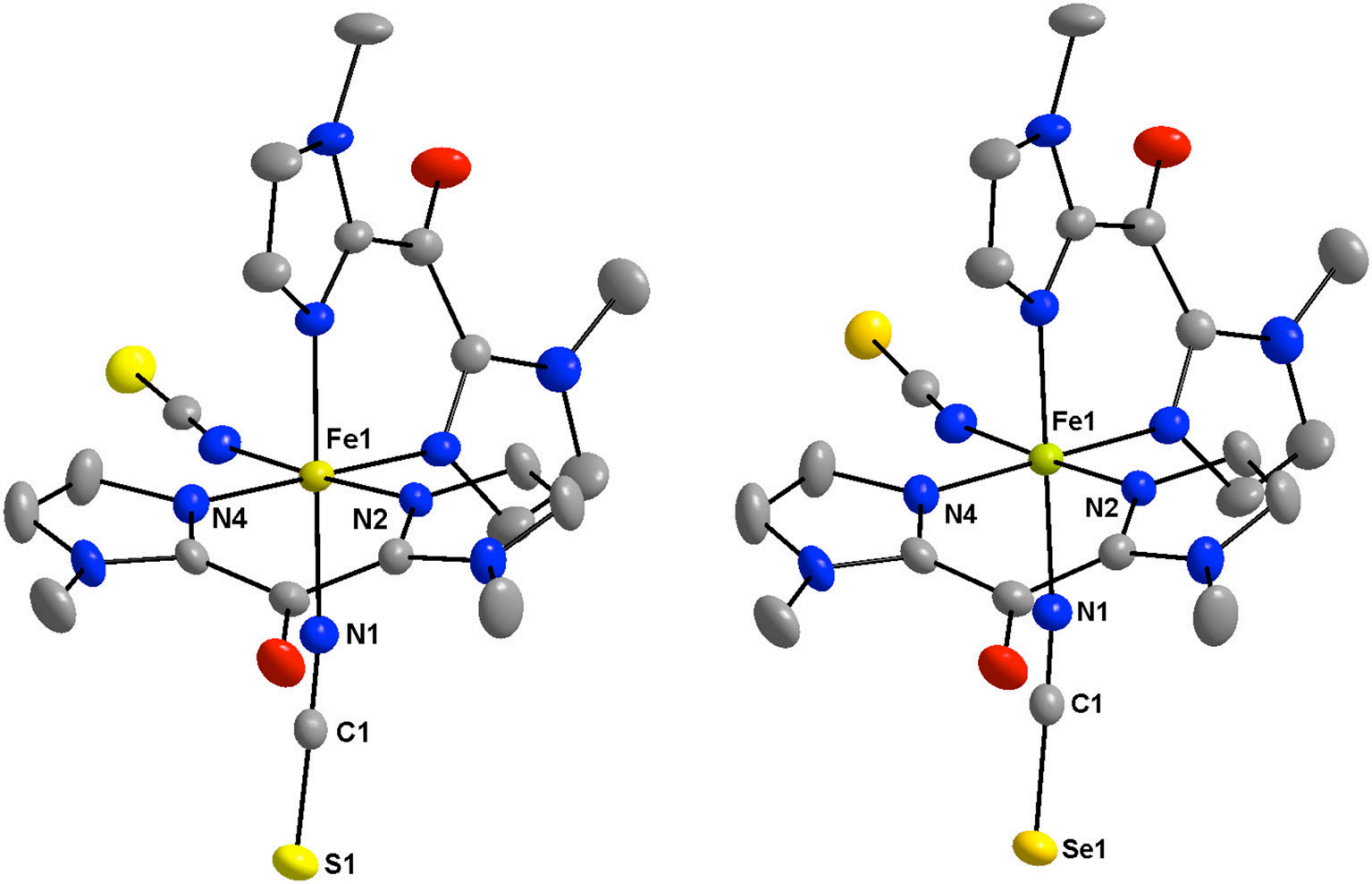
View of the low-spin crystal structure of 1 **(Left)** and 2 **(Right)** at 200 K with the atom numbering of the coordination sphere and the NCX ligands. All hydrogen atoms are omitted for clarity (Fe: golden, C: gray, N: blue, O: red; S: yellow, Se: orange).

**Figure 2:**
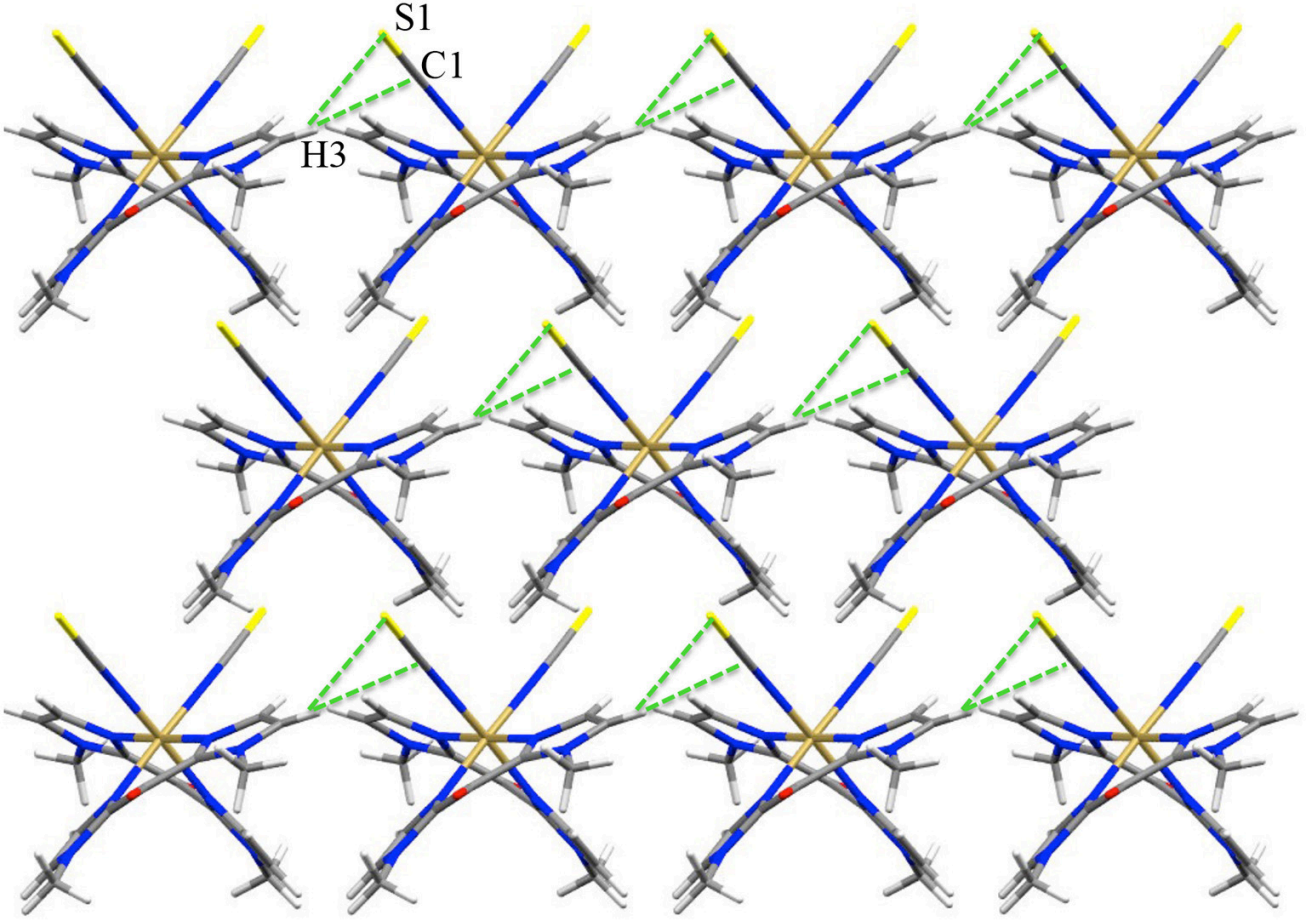
View of the pseudo H-bond interaction (dotted green lines) in 1 at 200 K (view along *c* axis).

The structures of **1** and **2** consist of mononuclear [Fe(^Me^bik)_2_(NCX)_2_] complexes (Figure 1), which are linked by Van der Waals interactions and pseudo-hydrogen bonds. No solvent molecules are observed in the crystal lattice, which is coherent with the TGA data (see Supplementary information). In both compounds, the asymmetric unit is made of half a complex, which is related to the other half through a *C*_2_ symmetry axis. A view of the crystal packing along the *c* axis is shown in Figure 2. In the complexes, the iron(II) atom is coordinated by two thio- or seleno-cyanate nitrogen atoms (for **1** or **2**, respectively), which are in *cis* position and four ^Me^bik nitrogen atoms, leading to a distorted octahedral [N_6_] coordination sphere. Selected interatomic distances and angles for **1** and **2** are listed in Tables S3, S4 and selected data of the coordination spheres for **1** and **2** are given in Table 2.

**Table 2:** Selected structural data of the coordination sphere in 1 and 2 (Distance are given in Å and angles in degrees).

	**[Fe(**^**Me**^**bik)**_**2**_**(NCS)**_**2**_**]**			**[Fe(**^**Me**^**bik)**_**2**_**(NCSe)**_**2**_**]**		
T (K)	200	300	400	200	300	400
< Fe-N(L)>^a^	1.967	2.141	2.169	1.960	1.989	2.140
Fe-NCX	1.967	2.108	2.133	1.961	1.974	2.132
< N-Fe-N>^a^ bite angle	88.98	84.03	83.08	89.11	88.11	83.74
[Fe-N-C(X)]_av_ angle	164.90	159.40	158.60	165.42	165.10	159.20
$\sum_{1}^{12}|90-\alpha |$^b^	23.41	33.80	37.26	23.36	26.64	34.74
τ^c^	0.0058	0.0317	0.0375	0.0075	0.0033	0.0083
Shape factor *S* (OC-6)	0.085	0.188	0.240	0.085	0.089	0.205

a *< Fe − N> and < N − Fe − N> are the averaged values over the different coordination bonds and bite angles, respectively*.

b *Sum of the deviation from the orthogonality of the twelve N-Fe-N angles*.

c *τ=Σ (Fe-N— < Fe − N>)*.

The comparison of the structures at different temperature points to the occurrence of a thermally-induced spin crossover in both compounds, which is also revealed by magnetometry measurements (*vide infra*). We examine first the structural data at 200 K.

The average Fe-N(NCS) and Fe-N(^Me^bik) bond lengths measured at 200 K are equal respectively in **1** (1.967 Å) and very similar in **2 (**respectively 1.959 and 1.962 Å). These distances are very homogeneous with an average deviation from the orthogonality of τ 0.006° (**1**) and 0.008° (**2**) (Table 2). These bond lengths are typical for low-spin octahedral Fe(II) complexes. For example, they are close to those previously reported in the low-spin [Fe(phen)_2_(NCS)_2_] (Real et al., 1992) and [Fe(bipy)_2_(NCS)_2_] (Konno and Mikami-Kido, 1991) complexes (Table 3). However in these last compounds, the bond deviation, τ, are higher because the Fe-NCX bonds are notably shorter. The distortion from the octahedral geometry in **1** and **2** is more apparent in the deviation from orthogonality of the twelve N-Fe-N angles involving the nitrogen donor atoms (see Table 2). The measured values, *ca*. 23°, are slightly above that measured in the low-spin [Fe(^Me^bik)_3_](BF_4_)_2_ complex (13.5°). The distortions of the Fe(II) coordination sphere were also analyzed by continuous shape measurements using the SHAPE program (Casanova et al., 2005; Llunell et al., 2005). The reported output (shape factor) allows assessing the matching between an idealized polyhedron and actual coordination sphere: the lower the shape factor is, the better the matching between the actual coordination sphere and the idealized polyhedron. The analyses lead to numerical values of 0.85 for both **1** and **2** at 200 K (0 would correspond to a perfect octahedron), a value which is a bit larger than that observed in the low-spin [Fe(^Me^bik)_3_](BF_4_)_2_ complex (0.42) (De et al., 2018).

**Table 3:** Metal-Ligand distances in related Fe(II) SCO complexes.

**Compounds**	**High temperature**		**Low temperature**		***ΔR*** **upon HS-to-LS conversion**		**References**
	**[Fe-NCX]**	**[Fe-N(L)]**	**[Fe-NCX]**	**[Fe-N(L)]**	***ΔR* [Fe-NCX]**	***ΔR* [Fe-N(L)]**	
[Fe(phen)_2_(NCS)_2_]	2.057	2.206	1.958	2.009	0.099	0.197	Real et al., 1992
[Fe(bipy)_2_(NCS)_2_]	2.053	2.173	1.945	1.966	0.108	0.207	Konno and Mikami-Kido, 1991
^a^[Fe(btz)_2_(NCS)_2_]	2.064	2.170	1.948	1.973	0.116	0.197	Real et al., 1992
[Fe(^Me^bik)_2_(NCS)_2_]	2.133	2.169	1.967	1.967	0.166	0.202	This paper
[Fe(^Me^bik)_2_(NCSe)_2_]	2.132	2.140	1.961	1.960	0.171	0.180	This paper
[Fe(phen)_2_(NCSe)_2_]	2.080	2.187	1.933	1.982	0.147	0.205	MacLean et al., 2003

a *btz = 2,2'-bi-4,5-dihydrothiazine; The [Fe-N] distances are averaged values*.

Concerning the geometry of the ligands, the β-di-imine ^Me^bik bidentate ligands are not planar in contrast with the bipy and phen α-di-imine ligands. However we showed in a previous study that the presence of the CO group between the two imidazolyl groups confers some aromaticity to the ^Me^bik ligand and thus, a π-acceptor character. (De et al., 2018) Here the dihedral angle between the imidazolyl rings are 24.9 (**1**) and 24.1° (**2**) at 200 K. These values are quite larger than those observed in the low-spin [Fe(^Me^bik)_3_](BF_4_)_2_ (between ca. 4 and 11°) (De et al., 2018). Finally the NCX ligands are linear (Tables S3, S4) however the Fe-N-C angle deviates significantly from linearity (*ca*. 165° in **1** and **2**) as previously observed in related [Fe(phen)_2_(NCX)_2_] complexes (Konno and Mikami-Kido, 1991; Real et al., 1992; MacLean et al., 2003).

The X-ray structure analyses carried out at higher temperature (300 and 400 K) reveal that complexes **1** and **2** undergo structural changes that are to be correlated with a spin-crossover process. The most obvious change is the significant increase of the metal-ligand bond lengths occurring upon heating. The average Fe-N coordination bonds increase by *ca*. 0.190 and 0.177 Å in **1** and **2**, respectively, between 200 and 400 K. Such an increase is within the expected range for a spin crossover occurring in a {${\text{Fe}}^{\text{II}}N_{6}$} system (Table 3) (Konno and Mikami-Kido, 1991; Real et al., 1992; MacLean et al., 2003). It is correlated to the increase of electronic population in the antibonding ${\text{e}}_{\text{g}}^{*}$ orbitals and the decrease of electronic population in the t_2g_ orbital, which decreases the π-backbonding on going from the LS to the HS state. It is worth noticing that the distance variations are larger for the Fe-N(^Me^bik) bonds than for the Fe-NCX ones. As suggested in previous studies, this may be correlated to the better π-acceptor character of the ^Me^bik ligand in comparison to the NCX (Konno and Mikami-Kido, 1991). However, it is not clear why this difference is less pronounced in the ^Me^bik complexes than in the α-di-imine complexes (Table 3). The significant increase of the Fe-N coordination bonds upon heating is also accompanied by an increase of the distortion in the coordination spheres, as revealed by the significant increase of the ∑ value and the S factor (Table 3). This behavior is characteristic of the conversion of low-spin Fe^II^ state to a high-spin Fe^II^ state.

The intermolecular interactions are also affected by the spin-state change, in particular, some intermetallic distances tend to decrease upon heating. Actually the analysis of the unit-cell parameters shows for both compounds a moderate thermal volumic expansion (>5%) that is related to an anisotropic and opposite variation of the axes. Beside the expansion along the b (5%) and c axes (1%), we observe a slight contraction along the *a* axis (1–2%). This contraction is correlated to the decrease of the smallest Fe^…^Fe distance (Table S5**)** between 200 and 400 K, from 7.774 to 7.681 Å in **1** and, from 7.916 to 7.849 Å in **2** (Table 1). This feature has already been observed in related [Fe(L-L)_2_(NCS)_2_] complexes (L-L are α-diimine ligands) and it is due to a scissor-like distortion of the complex during the spin-state change: the angle between the plans containing the L-L ligands decreases as the temperature increases (Shepherd et al., 2012).

In contrast, the pseudo hydrogen bonds between the S or Se atoms and a hydrogen atom of a ^Me^bik ligand belonging to a neighboring complex (Figure 2, Supplementary Figure S1), increase when the temperature increases (Table 4). This might be correlated to the decrease of the Fe(1)-N(1)-C(1) angle (*ca*. 6° in both **1** and **2**) between 200 and 400 K. This angle variation might allow in part the absorption of the strain caused by the increase of Fe-N bond lengths accompanied by the spin-transition (Pap et al., 2011a,b,c; Scheja et al., 2015). The bending also indicates that the Fe-N = C = X mesomeric form (X = S or Se) coexists with the Fe-N=C-X^−^, which is expected to be linear. This is also reflected in the C-S and C-Se bond distances, which are intermediate between a double and a triple bond (*ca*. 1.62 and 1.78 Å respectively).

**Table 4:** Pseudo-hydrogen bonds (Å) in 1 and 2.

	**r(vdw)^a^**	**200(2)**	**300(2)**	**400(2)**
S(1)—H (7)	3.00	2.85	2.94	2.97
Se(1)—H (7)	3.10	2.96	2.99	3.02

a *Sum of van der Waals radii*.

Overall the structural changes in the coordination sphere of **1** and **2** point to a full (or almost complete) spin crossover between 200 and 400 K. However the main structural changes occur between 200 and 300 K in **1** whereas in **2** they occur between 300 and 400 K. These structural data indicate that the spin transition in **1** should be centered at a lower temperature (*ca*. < 300 K) than that in 2 (*ca*. > 300 K), which is consistent with the weaker ligand field induced by the NCS ligand as compared to the NCSe one (Nakano et al., 2004; Ross et al., 2011; Klingele et al., 2012).

### Optical properties

**Figure 3:**
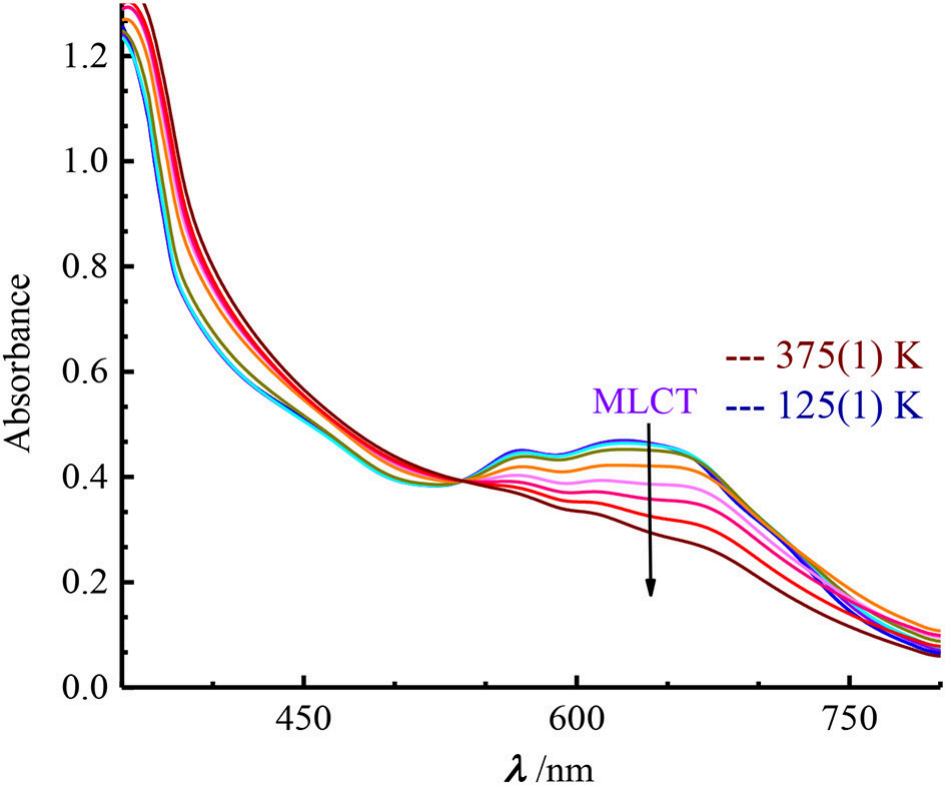
Temperature dependence of the UV-Vis spectra of 1 between 125 and 375 K.

The solid-state absorption spectra were collected on crystallites of the compounds dispersed in KBr pellets within a temperature range of 125–375 K. Similarly to the [Fe(^Me^bik)_3_]^2+^ spin-crossover complex, **1** and **2** exhibit an asymmetric broad absorption band centered near 615 nm, with several contributions included those centered at ~ 570 and 655 nm (**1**) and 550 and 650 (**2**) (Figure 3 and Supplementary Figure S2). These bands responsible for the deep blue color of the complexes are ascribed to metal-to-ligand charge transfer (MLCT) absorption as confirmed by TD-DFT calculations (see below). Upon heating, the spectral evolution is similar in both compounds: (i) a pseudo isosbestic point appears; (ii) a decrease of intensity and a high-energy shift of the MLCT absorption are observed. These features match to the trends generally observed in this class of spin-crossover complexes. (Mondal et al., 2014; De et al., 2018) In fact, the larger metal-ligand bond lengths in the high-spin Fe^II^ complexes result in a weaker overlap between metal-centered and ligand-centered orbitals as compared to the low-spin state. Therefore, the intensity of the MLCT band strongly decreases upon converting the LS into HS species.

The intense bands observed below 450 nm are ascribed to intra-ligand charge transfer transitions. On the low energy side of the spectrum, no other bands were clearly evidenced in the solid-state but a very weak unresolved absorption is observed in solution (see Supplementary Figures S2, S3). This weak band could be due to a ligand-field transition.

### Magnetic properties

**Figure 4:**
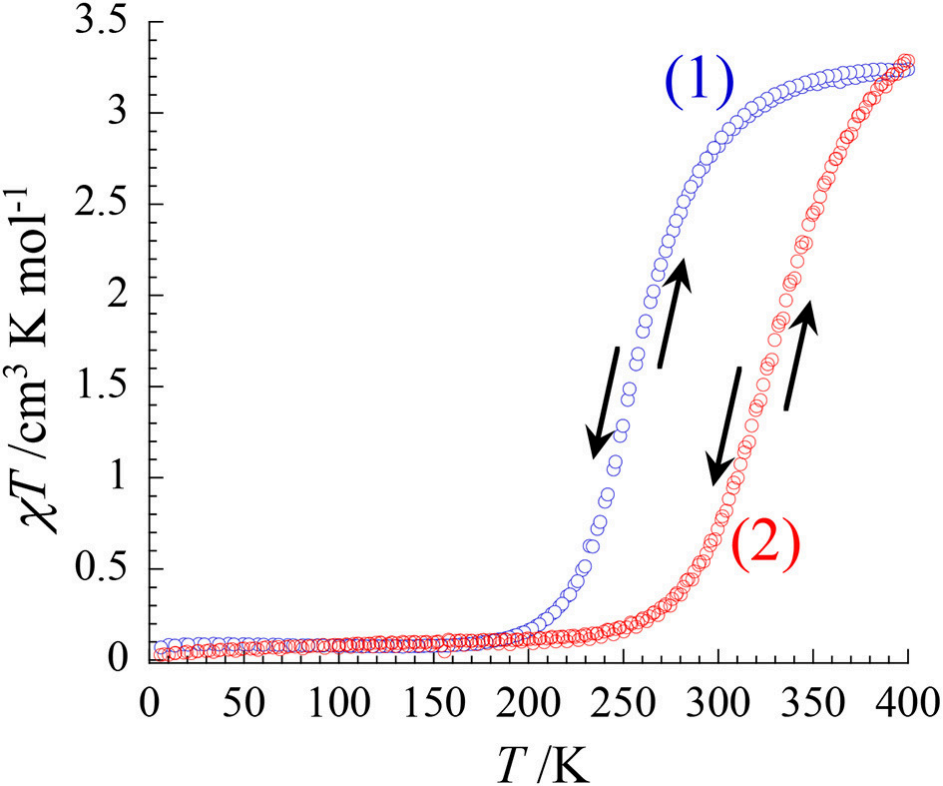
Temperature Plot of χ*T* vs. *T* for 1 and 2 under an applied magnetic external field of 1 T.

The magnetic properties of both complexes were investigated in the 4.0–400 K range by measuring the temperature dependence of the χ*T* product (χ being the molar magnetic susceptibility) upon heating and cooling (at 2 K/min). The χ*T* curves of **1** and **2** exhibit sigmoidal shapes, which are typical of the occurrence of a spin-crossover. In **1**, the transition occurs between 170 and 380 K with an estimated transition temperature *T*_1__/2_ of *ca*. 250 K. The significant variation of the χ*T* value (from 0.08 cm^3^ mol^−1^ K at 150 K to 3.25 cm^3^ mol^−1^ K at 400 K) points to a complete thermally-induced S = 0 (${\text{t}}_{2\text{g}}^{6}$) ⇔S = 2 (${\text{t}}_{2\text{g}}^{4}$${\text{e}}_{\text{g}}^{2}$) spin crossover. The magnetic behavior of **2** follows a similar trend but the spin transition starts at higher temperature (*ca*. 240 K) and seems almost complete at 400 K. The χ*T* value increases from *ca*. 0.10 cm^3^ mol^−1^ K at 200 K up to *ca*. 3.30 cm^3^ mol^−1^ K at 400 K. The estimated transition temperature is *ca*. *T*_1__/2_ ≈ 330 K (Figure 4).

Overall these magnetic data agree well with the structural investigation at different temperature and the optical spectroscopic studies at variable temperature. The thiocyanate derivative (**1**) exhibits a LS ⇔ HS spin transition, occurring below room temperature whereas the observed transition in selenocyanate derivative is shifted above room temperature. This is coherent with a stronger ligand field in the [Fe(^Me^bik)_2_(NCSe)_2_] complex than in the [Fe(^Me^bik)_2_(NCS)_2_] one. This agrees well with previous studies on related α-diimine-based complexes such as [Fe(phen)_2_(NCS)_2_] and [Fe(phen)_2_(NCSe)_2_] (Real et al., 1992; MacLean et al., 2003).

In these compounds, the spin transition was quite abrupt, whereas in the present case the transition is much more gradual. This difference can be explained by the weaker intermolecular interactions involved in **1** and **2** as compared to those observed in [Fe(phen)_2_(NCX)_2_], which show π-π interaction beside the pseudo hydrogen bonds (Real et al., 2003; Gütlich et al., 2005) (MacLean et al., 2003).

The cooperativity between the spin-crossover complexes in the crystal lattice of **1** and **2** can be quantified by analyzing the χ*T* vs. *T* curves through a regular solution model (expressed by the following equation): (Slichter and Drickamer, 1972).

(1) $$\begin{array}{l} \ln\;[(1-n_{HS})/(n_{HS-}f_{HS})=\\ \;[\Delta H+\Gamma (f_{HS}+1-2n_{HS})]/RT-\Delta S/R \end{array}$$

where Δ*H* and Δ*S* are the enthalpy and entropy variation induced by the spin transition, Γ parameter is the cooperativity factor associated with the spin crossover (*f*_HS_ is the residual HS molar fraction at low temperature and *n*_HS_ represents the HS molar fraction).

The analyses lead to ΔH = 22.2 (**1**) 20.1 kJ mol^−1^ (**2**), ΔS = 85.3 (**1**) 61.7 (**2**) J K^−1^ mol^−1^, and Γ = 1.5 (**1**) 3.3 (**2**) kJ mol^−1^, respectively. The enthalpy and entropy variations are somehow lower than those obtained in the [Fe(^Me^bik)_3_](BF_4_)_2_ complex but they remain in the expected range for Fe(II) SCO complexes. ((Nakamoto et al., 2001; Martínez et al., 2009); (Scott et al., 2013; Kumar et al., 2015)).

In contrast, the Γ values are moderate but higher in **1** and **2** than in the [Fe(^R^bik)_3_](BF_4_)_2_ complexes where only very weak intermolecular interaction were found. The larger value measured in **2** agrees well with the crystallographic data mentioned above, which revealed a stronger pseudo H-bond in compound **2** in comparison to compound **1**. Finally, it is worth noticing that both spin transition are perfectly reversible: the χ*T* vs. *T* curve obtained upon cooling superimposed perfectly with that measured upon heating. The absence of solvent molecule in the crystal lattices of **1** and **2** likely accounts for this reversibility.

### The photomagnetic behavior

**Figure 5:**
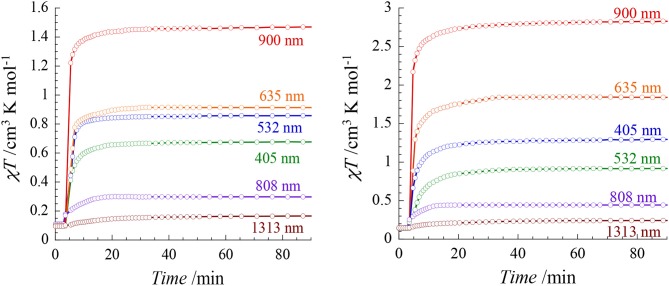
χ_M_*T* vs. irradiation time of 1 **(Left)** and 2 **(Right)** measured at 20 K for different wavelengths (*ca*. 5 mW/cm^2^).

The photo-sensitivity of both compounds was investigated at 20 K by using laser diodes (with a power of *ca*. 7 mW/cm^2^) in the range 400–1,300 nm (405, 532, 635, 808, 900, 1,313 nm). Both compounds show Light-Induced Excited Spin-State Trapping (LIESST) effect in almost the all energy range (Figure 5). However significant differences are observed depending on the irradiation wavelengths. In both cases, the strongest effect is obtained at 900 nm. LIESST effects involving near IR irradiations are not so common, however some examples have been reported (Glijer et al., 2008; Buron-Le Cointe et al., 2012; Marino et al., 2014). Irradiation at 635 nm in the center of the MLCT band also leads to a LIESST effect but with a lower conversion that could be due to a smaller light penetration. Surprisingly, the effect of the 808 nm laser diode is remarkably low and it leads to a very poor conversion as compared to the irradiation at lower (900 nm) and higher (635 nm) energy.

The relative efficiency of the different wavelengths is similar in both compounds, however the conversion rates are overall higher in **2** than in **1**. In particular the irradiation at 900 nm leads to an almost quantitative spin conversion in **2**: the χ_*M*_*T* value reaches 2.83 cm^3^ mol^1^ K after 30 min in comparison to 1.46 cm^3^ mol^−1^ K in **1**. When comparing these results to those obtained on our previously reported [Fe^II^(^Me^bik)_2_(N-)_2_] complexes, we observe that the nature of the N- donors groups allows to tune the energy of the most efficient wavelength promoting the LIESST effect. In the [Fe(^Me^bik)_3_](BF_4_)_2_ complex, the optimal LIESST effect is observed at 635 nm (De et al., 2018). When the N- donors are the metallo-ligands, [Mo(CN)_8_]^3−^ or [Fe(Tp)(CN)_3_]^−^ (in the cyanide-bridged rhombus molecules of formula {[Mo(CN)_8_]_2_[Fe(^Me^bik)_2_]_2_}(^H^MeIm)_2_·5H_2_O·CH_3_CN, where ^Me^ImH^+^ = N-methyl-imidazolium cation, and {[Fe_III_(Tp)(CN)_3_]_2_[Fe^II^ (^Me^bik)_2_]_2_}.[Fe^III^(Tp)(CN)_3_]_2_·18H_2_O·4CH_3_OH), the most efficient effect are observed at 405 and 808 nm, respectively (Mondal et al., 2013, 2014) These differences should be related to the different electronic structures of the compounds and the nature of the accessible excited-states involved in the LIESST effect. The occurrence of reverse LIESST effect in some of these compounds was also proposed to account for a partial decrease of the photomagnetic response (Moussa et al., 2007). The rationalizing of the wavelength dependence on the LIESST effect would be interesting but it requires a set of advanced physical measurements that is beyond the scope of the present report.

It is also worth noting that the relaxation temperature, *T*_*LIESST*_, are significantly higher (*ca*. 60 K) in all the previously reported complexes based on the [Fe^II^(^Me^bik)(N-)_2_] units in comparison to those observed in **1** and **2**. Here, both complexes exhibit a rapid relaxation if the light is switched off, revealing that the *T*_*LIESST*_ are below 20 K. This unusual behavior might be associated to the existence of a different relaxation process in **1** and **2**. Recently, Heinze and McCusker showed that the existence of low-lying ^3^T_1_ state might open efficient relaxation pathway from the MLCT state to the ^1^A_1_ ground state in Fe^II^ complexes containing pyridine-based ligand connected by keto groups (Jamula et al., 2014; Mengel et al., 2015).

### Theoretical calculations

As observed in our earlier studies on the [Fe(^R^bik)_3_](BF_4_)_2_ complexes featuring spin crossover properties(De et al., 2018), the choice of a suitable exchange-correlation functional within DFT method which could accurately predict both the ground state structure and SCO characteristics is challenging. For example, although the spin-state energies (Δ*E*_*HS*−*LS*_) is in accordance with the SCO properties, hybrid B3LYP functional fails to correctly predict the ground state. In contrast, a modified version where percentage Hartree-Fock (%HF) exchange is scaled to 15% (B3LYP^*^) and OPBE predict the ground state correctly but the predicted spin-state splitting are too high to suggest SCO characteristics. Cooperative effects in SCO molecules play a very important role and there are methods available within the DFT to estimate such effects comprehensively (Rackwitz et al., 2013; Scott et al., 2013; Paulsen, 2016). In this present work, we have probed the energetic of spin-state rearrangements and the excited state properties within the intramolecular framework. The PCM (Tomasi et al., 2005) solvent model was employed to counter the effects of solvent molecules around the metal complex. While this qualitatively address the SCO effect in solution, the effect observed in solid-state are still missing.

**Figure 6:**
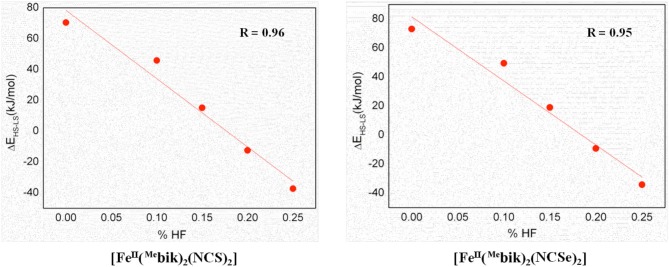
DFT computed spin-state energies (Δ*E*_*HS*−*LS*_) vs. HF exchange percentage employing BLYP functional for complexes 1 and 2.

As %HF exchange is found to play a role in dictating the spin-state splitting, we decided to vary the %HF exchange in B3LYP to assess and understand how this influence the SCO characteristics of [Fe(bik)_2_(NCX)_2_] complexes. Additionally for comparative purpose, calculations are also performed using OPBE functional. The optimized energies of complexes **1** and **2** (Table 5) reveal that the B3LYP functional predicts a high-spin ground state in both the cases, whereas all other variation attempted (BLYP, B3LYP^*^, OPE, etc.) suggest a low spin as the ground state. The difference in energy between the high-spin and the low-spin state defined as Δ*E*_*HS*−*LS*_ is computed varying the % HF exchange using BLYP functional. A linear dependency of Δ*E*_*HS*−*LS*_ on the %HF exact exchange is observed for both complexes **1**-**2** (Figure 6) as reported earlier for other SCO complexes. (Grimme, 2006; Bowman and Jakubikova, 2012; Kepp, 2013) The Δ*E*_*HS*−*LS*_ energies in case of B3LYP and BLYP (with 25%HF exchange) are estimated to be −12.5 and −37.3 kJ/mol in **1**, and −9.2 and −34.2 kJ/mol in **2**. Calculations reveal that as the % HF exchange increases, the Δ*E*_*HS*−*LS*_ gap decreases with HS becoming ground state from 20% HF onwards. The Δ*E*_*HS*−*LS*_ energies summarized in these cases as, 15.1 (15%), 45.8 (10%), and 70.4 (0%) kJ/mol in **1**, whereas the same estimates are 19.0 (15%), 49.5 (10%), and 73.1 (0%) kJ/mol in **2**. On the other hand, OPBE functional estimated the Δ*E*_*HS*−*LS*_ energies are, 14.0 and 19.2 kJ/mol for complexes **1** and **2**, respectively. It is important to note that the Δ*E*_*HS*−*LS*_ energies are slightly higher in **2** as compare to complex **1**. This is in agreement with the greater covalency associated with the selenium atom as compare to sulfur. Lastly the computed energies using B3LYP (%HF = 20), B3LYP^*^ (%HF = 15) and OPBE functional estimates the Δ*E*_*HS*−*LS*_ gap below 20 kJ/mol for both **1** and **2**. These values are coherent with the occurrence of a spin-crossover phenomena (Ye and Neese, 2010b) in both complexes (**1** and **2**) and in line with the experimental observations where transition are measured at *T*_1/2_ of 260 and 326 K for **1** and **2**, respectively.

**Table 5:** DFT computed Δ*E*_*HS*−*LS*_ (*in* kJ/mol) obtained with different %HF exchange values (0–25%) using BLYP and including OPBE.

	**HF−0%**	**HF−10%**	**HF−15%**	**HF−20%**	**HF−25%**	**OPBE**
**1**	70.4	45.8	15.1	−12.5	−37.3	14.0
**2**	73.1	49.5	19.0	−9.2	−34.2	19.2

To understand how temperature influences the geometrical parameters, we have performed geometry optimization starting from the X-ray structures of **1** and **2** obtained at different temperatures. Due to the difference in the *T*_1/2_ observed, it is clear from the estimated structural parameters that the high-spin optimized structure at 300 K of **1** is identical with the one computed at 400 K, while the 300 K structure in case of **2** is identical with the low-spin optimized structure at 200 K (Table 2). Here the optimized spin-state energies (Δ*E*_*HS*−*LS*_) of **1** and **2** are considered for discussion.

**Figure 7:**
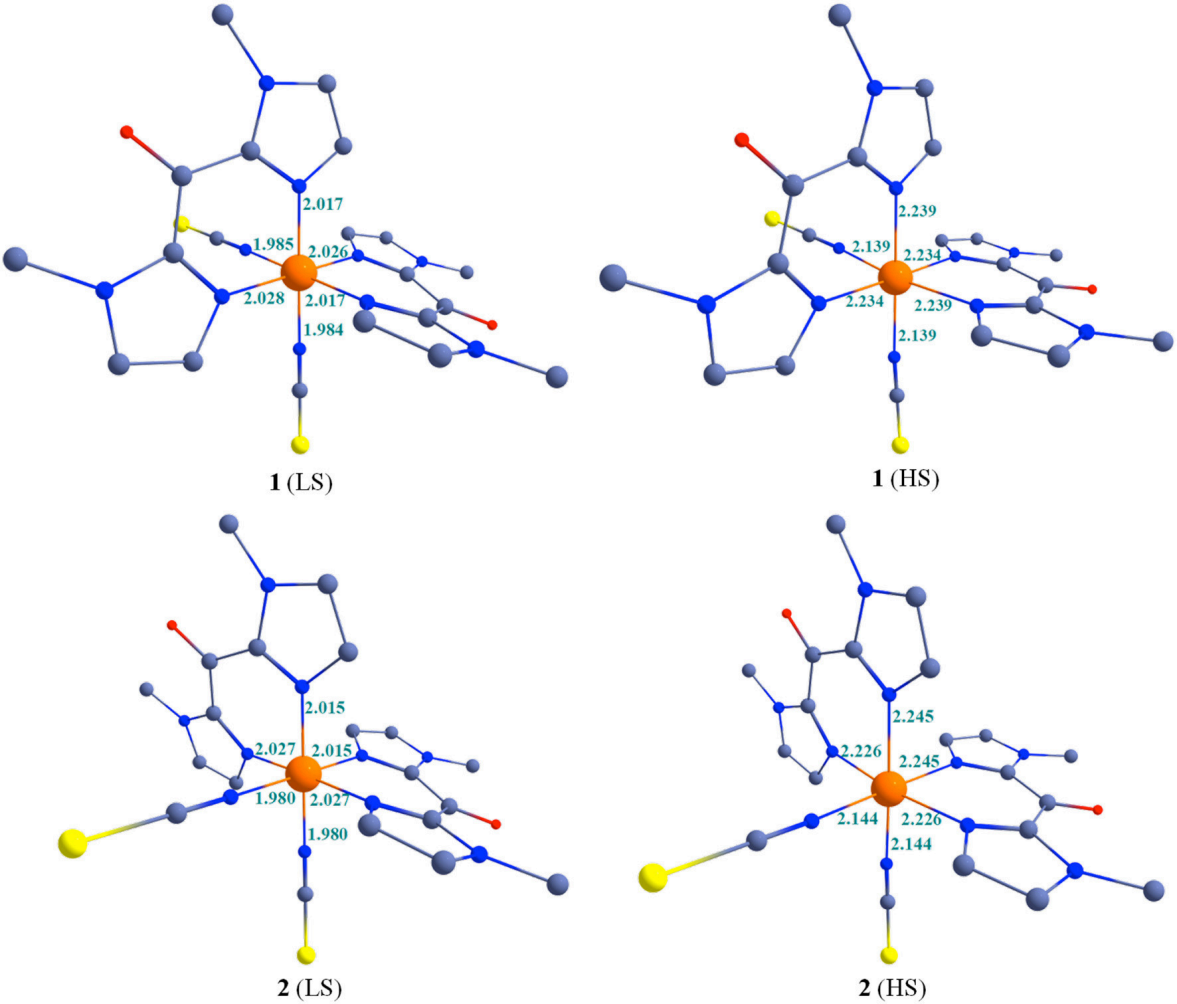
B3LYP optimized structures along with some selected bond parameters of complexes 1-2 at 200 and 400 K. Colour code: Blue = N; Red = O; Yellow = S/Se; Gray = Carbon. Hydrogen atoms are omitted for clarity.

The optimized geometries of the low-spin and high-spin geometries of **1** and **2** are shown in Figure 7 and some selected bond parameters are summarized in Table 6. The choice of DFT functional plays an important role on the optimized bond parameters. Here using B3LYP functionals, metal-ligand bond distances (in the structure optimized in gas phase at 0 K) are overall in very good agreement with the experimental values (Table 2). They appear slightly overestimated as compare to the experimental X-ray structures, in particular for the Fe-N(bik) distances. The significant increase of the Fe-N bond length upon heating is well reproduced by the theoretical calculation. In fact the bond distance variation associated with the Fe-N(bik) and Fe-NCX bonds are estimated to be 0.22 and 0.15 Å in **1**, and 0.21 and 0.16 Å in **2**, respectively. These values are close to the experimental ones (Table 2), in particular, the larger variation in the Fe-N(bik) bond lengths as compare to the Fe-NCX bonds is also observed in the optimized structure. This further supports the fact that the ^Me^bik ligands offer suitable π-acceptor pathway as compare to the NCX ligand.

**Table 6:** Selected bond parameters based on B3LYP optimized geometries for 1 and 2.

**B3LYP**	**[Fe**^**Me**^**(bik)**_**2**_**(NCS)**_**2**_**]**		**[Fe**^**Me**^**(bik)**_**2**_**(NCSe)**_**2**_**]**	
**T (K)**	**LS**	**HS**	**LS**	**HS**
*d*[Fe-N(L)]_av_ (Å)	2.022	2.237	2.021	2.235
*d*[(Fe-NCX)]_av_(Å)	1.985	2.139	1.980	2.144
axial (N-Fe-N)_av_ angle (°)	179.4	172.9	179.9	175.3
bite (N-Fe-N)_av_ angle (°)	87.9	86.6	87.9	86.9
[Fe-N-C(X)]_av_ angle (°)	178.2	174.2	178.7	175.8

The distortion of the octahedral coordination sphere observed in the experimental X-ray structures is also reflected in the calculated ones. For example, the computed axial (N-Fe-N)_av_ angles decrease by ca. 5° between the LS and HS states, as observed in the experiment (Table 2). It is worth noting that optimized geometries are more symmetric compare to the X-ray structures for **1** and **2**. For example the average axial and bite (N-Fe-N)_av_ bond angles in the computed structures are close to the 180° and 90°, respectively. This reveals that solid-state effects contribute to the distortion in the coordination sphere in the actual structure. This is particularly clear for the bending of the NCX ligands, which are involved in pseudo hydrogen interaction in the crystal lattice: whereas the computed Fe-N-C angle is close to linearity, the experimental ones vary between ca. 165° (LS state) and ca. 159° (HS state).

### Thermodynamic parameters and molecular orbital analysis

Thermodynamic parameters estimated from the frequency calculations on top of the optimized coordinates are summarized in Table 7. The contribution from vibrational modes is factored in the energy as zero-point corrected energies are used for this purposed. The high-spin state is always favored by the vibronic entropy; i.e., strong field ligands that energetically favors the low-spin state suffers larger loss to the vibronic entropy. The estimates of the calculated entropy (Δ*S*) and enthalpy (Δ*H*) are 88.3 and 84.9 J/mol-K, and −18.0 and −14.2 kJ/mol (B3LYP) for **1**-**2**, respectively. These estimates of the calculated entropy are on a higher note but in fair agreement with the experimental data derived from the magnetic measurements (see above). The calculated Δ*H* values are slightly underestimated as compared to the experimental data obtained on the solid-state measurements. They are also relatively smaller as compare to the estimates from [Fe(^R^bik)_3_](BF_4_)_2_ complexes (De et al., 2018), and these further manifests that the ^R^bik ligand exerts stronger ligand fields as compare to the NCX (X = S/Se) ligand due to relatively stronger π-acceptor abilities. The OPBE calculated values of Δ*S* and Δ*H* parameters follows the footsteps of the B3LYP calculated results whereas the B3LYP^*^ computed results are unsatisfactory. The differences in the entropy (TΔ*S*) and the enthalpy (ΔH) contributions sum up to lead to the difference in the values of free energy component (Δ*G*_*el*_), 43.9 kJ/mol and 39.3 kJ/mol for **1** and **2** respectively. Calculated Δ*E*_*vib*_ values on the other hand are on the range of typically iron(II) octahedral complexes as observed earlier (De et al., 2018).

**Table 7:** Comparison of the calculated thermodynamic parameters of 1 and 2.

**Δ = HS – LS^[b]^**	**[Fe(bik)**_**2**_**(NCS)**_**2**_**]**			**[Fe(bik)**_**2**_**(NCSe)**_**2**_**]**		
	**B3LYP**	B3LYP^*^	**OPBE**	**B3LYP**	B3LYP^*^	**OPBE**
Δ*H_*el*_* (kJ /mol)	−18.0	62.3	12.9	−14.2	70.7	12.9
Δ*H_*el*_* ^[a]^ (kJ /mol)	−20.1	7.1	7.9	−10.0	35.1	12.9
Δ*G_*el*_* (kJ /mol)	−43.9	53.9	−17.1	−39.3	39.7	−13.1
Δ*G_*el*_* ^[a]^ (kJ /mol)	−42.8	−15.1	−22.6	−33.4	11.7	−13.1
Δ*E_*vib*_*(kJ/mol)	−5.4	−7.9	−6.2	−5.0	−3.7	−6.2
Δ*E_*vib*_*^[a]^ (kJ/mol)	−8.4	−8.8	−5.8	−3.3	13.8	−6.2
Δ*S* (J/mol-K)	88.3	88.1	101.7	84.9	103.7	101.6
Δ*S*^[a]^ (J/mol-K)	74.5	74.9	102.5	78.6	79.5	101.6

[a] *B3LYP-D2 dispersion corrected values*;

[b] *difference between the HS and LS values computed are listed here*.

**Figure 8:**
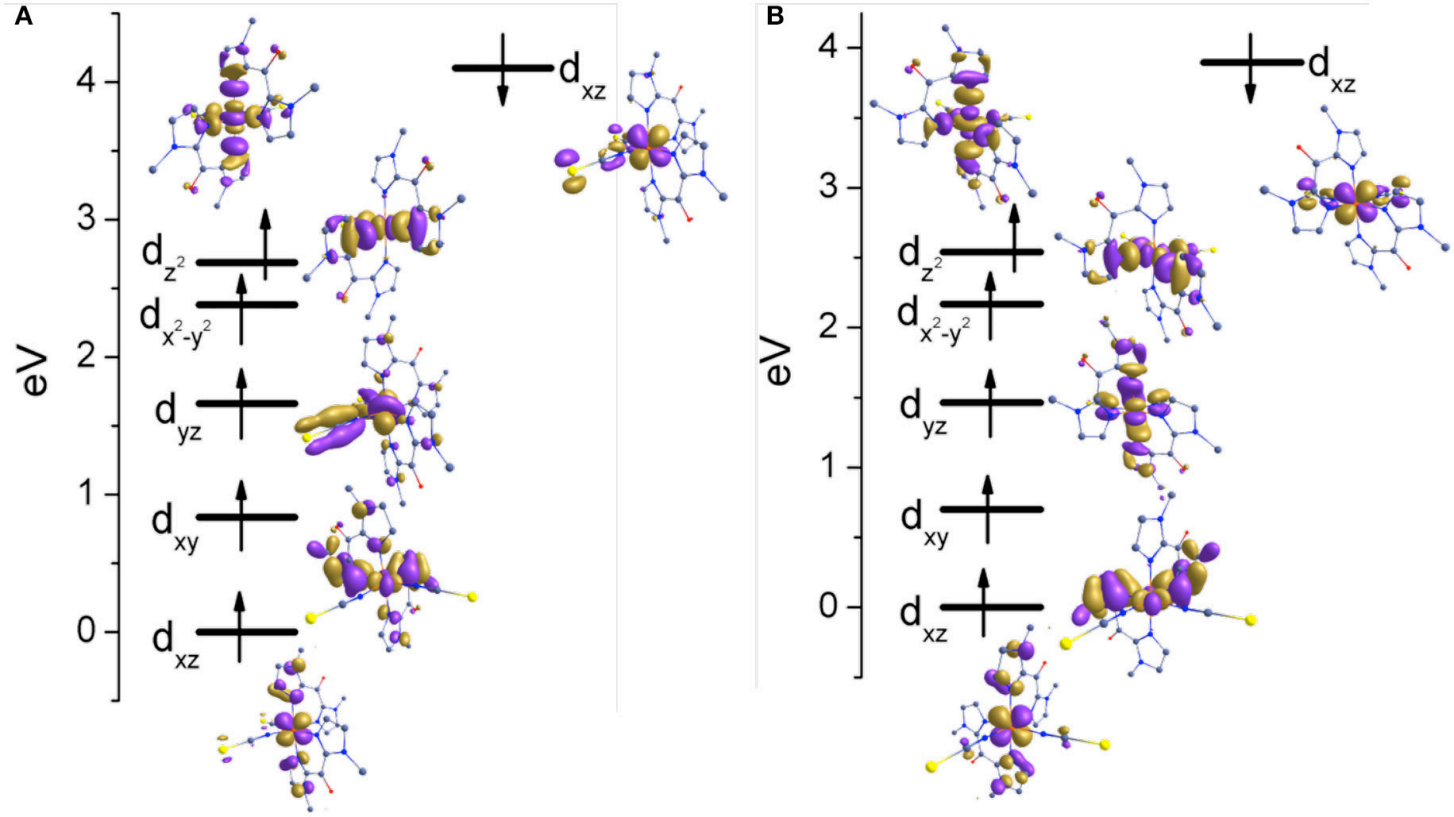
Energy splitting of d-based orbital's for **(A)** complex 1 **(B)** complex 2 in the high spin structures.

The nature of the spin ground state is determined by the orbital splitting and the spin pairing energy. Therefore, to gain further insights into the nature of the bonding, the d-based orbitals of complexes **1** and **2** at their respective high-spin state (400 K) were plotted using the eigenvalue orbital energies (Figure 8). For **1** and **2** HS structures, the B3LYP calculated electronic configuration yields the following, (*d*_xz_)^2^(*d*_xy_)^1^(*d*_yz_)^1^(*d*_x2−y2_)^1^(*d*_z2_)^1^. The splitting of d orbitals is estimated to be 2.685 and 2.541 eV for complexes **1** and **2**, respectively. These values are much higher as compare to [Fe(^R^bik)_3_](BF_4_)_2_ (~1.5 eV) complexes (De et al., 2018). Beside, the t_2g_ set of bonding orbitals are non-degenerate in **1** and **2** in contrast with the previous observations. This is due to the different σ-bonding interaction pattern of the ^R^bik and the NCX ligands. The splitting patterns for complexes **1** and **2** are consistent with each other. There is a slight difference in the energy of crystal splitting due to the greater covalency of the metal-ligand bonds in the selenium derivative as compare to the sulfur one. The extended delocalization of π-clouds from the d_xz/yz_ orbitals of the iron(II) center to the imidazole group is significantly high in the NCSe analog (**2**) as compare to the NCS analog (**1**) which can also be identified from the shorter Fe-N(^Me^bik) bond distances and the larger computed Δ*E*_*HS*−*LS*_ energy gap. Finally, it is worth noticing that there is no C-H^…^O interactions between the methyl hydrogen and the ketone oxygen atoms, in contrast with the situation in the [Fe(^R^bik)_3_](BF_4_)_2_ complexes (De et al., 2018). This is correlated to the weaker π-bonding interaction in [Fe(^R^bik)_2_(NCX)_2_] complexes as compare to the [Fe(^R^bik)_3_](BF_4_)_2_ analogs. Hence the hydrogen atoms of the C-H bond of the methyl group in complexes **1** and **2** are less polarized to promote such interaction.

**Figure 9:**
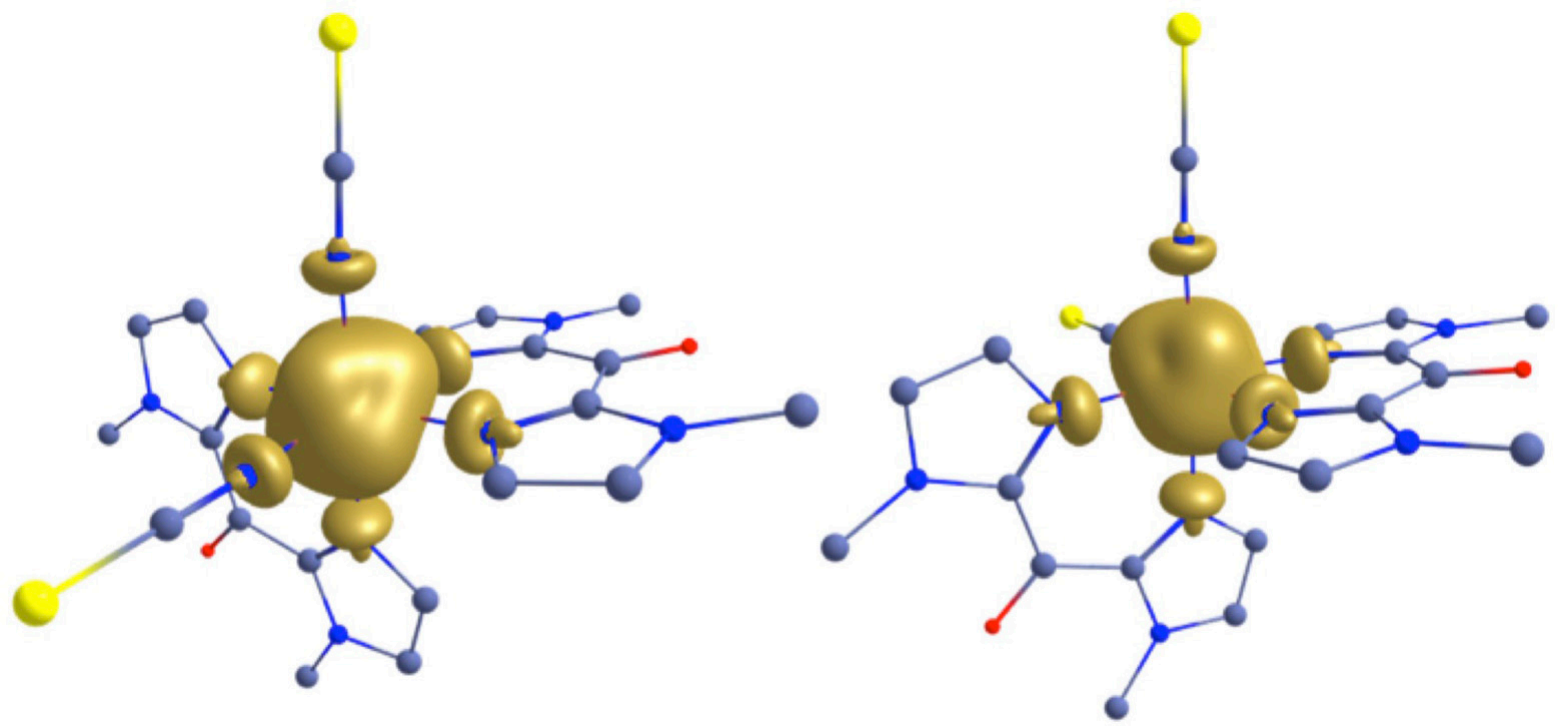
B3LYP computed spin density plot for 1 (left) and 2 (right) in the high spin geometry.

The spin density calculations also reflect somehow the bonding interaction in the complexes. The iron(II) center of **1** and **2** high spin structures shows almost identical spin density values of 3.830 and 3.826, respectively (Figure 9). The smaller spin-density value of the iron(II) in **2** reflects the relatively larger delocalization (back bonding) as compare to **1**. The net spin densities at the N-donor center for N-bik and NCX ligand are 0.020 and 0.013 for both **1** and **2**, respectively. The larger spin densities at the N-bik reflects the greater π-acceptor ability of the organic ligand as compare to the NCX ligands. Although the plotted spin density isosurface of complex **1**-**2** and [Fe(^R^bik)_3_](BF_4_)_2_ complexes (De et al., 2018) are nearly identical and the quoted spin density values are small, the difference observed between the two set of complexes clearly indicates significant structural and electronic alteration upon ligand modifications owing to variations in the SCO properties.

### Optical properties

**Figure 10:**
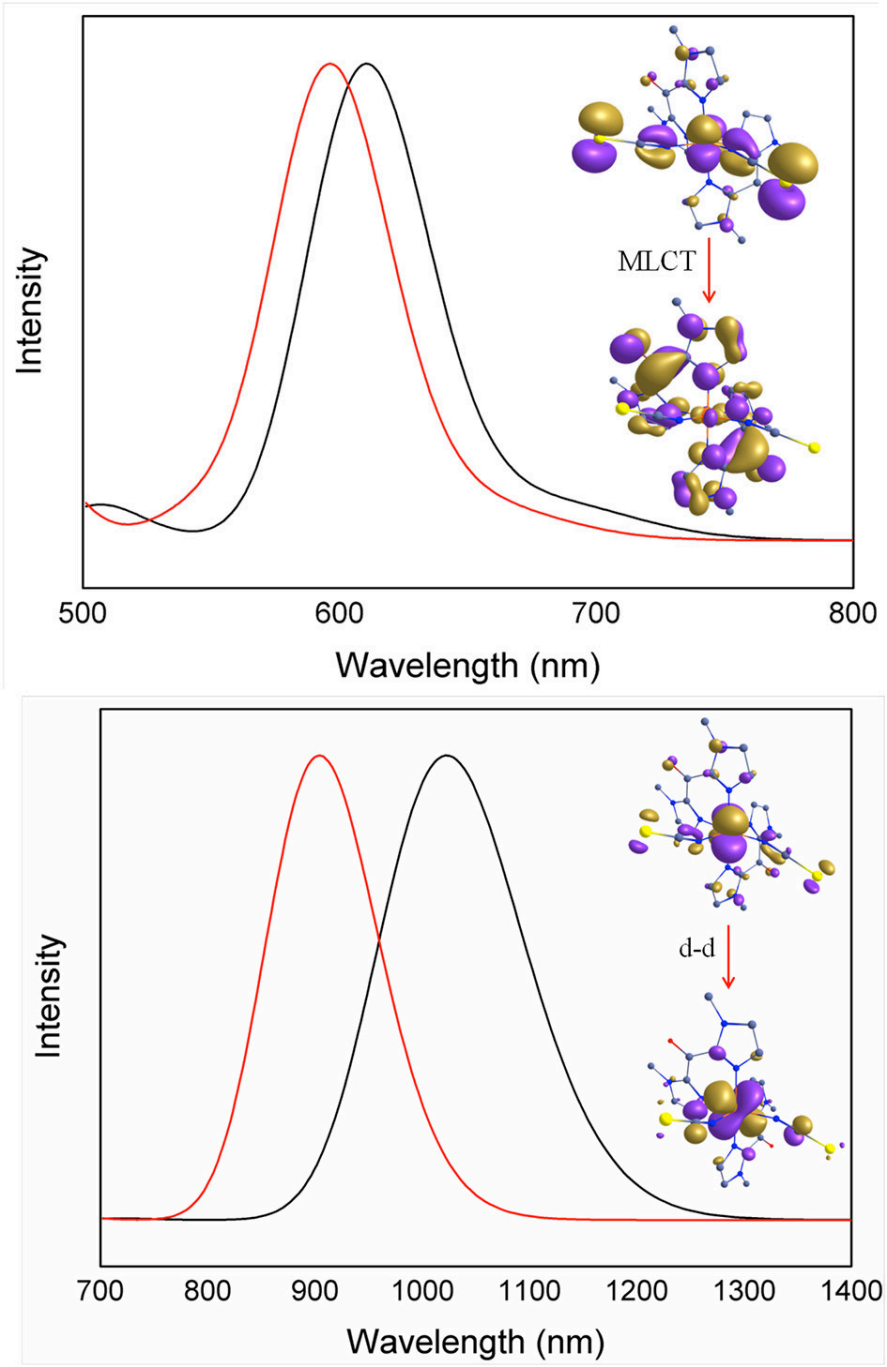
TDDFT computed absorption spectra of complexes low-spin state **(Top)** and high-spin state **(Bottom)** of 1 (red) and 2 (black) depicting important electronic transitions.

In order to rationalize the optical properties of complexes **1** and **2**, TD-DFT calculations using B3LYP functional have been performed on the X-ray structures. The computed absorption spectra are shown in Figure 10, Supplementary Figure S4. Calculated spectra of complexes **1** and **2** in the low-spin state show intense bands near *ca*. 611 nm and 596 nm, respectively, which are consistent with the experimentally observed intense band that were presumably assigned to MLCT bands (see above). Actually the theoretical calculations reveal that these bands consist of a series of transitions LL'CT and MLCT as it has been noted earlier in other Fe(II) low-spin complexes. (Mengel et al., 2015) The different contributions of the absorption spectra of **1** and **2** are listed in Table 8 and the corresponding orbital diagrams are given in Supplementary Figure S6 in ESI.

**Table 8:** Computed absorption spectra for complexes 1 and 2 along the various contributions to these transitions and their assignments (see Supplementary Figure S6 in ESI for orbital diagram).

	**λ (nm)**	**E (cm^−1^)**	**Osc. Strength**	**Nature of the transition^a^**
High-spin [Fe^II^(^Me^bik)_2_(NCS)_2_]	298 322 402 488 904	33616 31089 24897 20495 11062	0.374 0.031 0.000 6.83E-05 0.029	π -π^*^ (N^bik^ → N^bik^) LMCT (NCS → Fe) MLCT (Fe → N^bik^) LL'CT (NCS → N^bik^) d-d (Fe d_xz_ → Fe d_yz_)
Fe^II^(^Me^bik)_2_(NCSe)_2_]	320 340 435 588 1023	31256 29407 22981 16997 9777	0.029 0.054 0.005 0.006 0.084	MLCT (Fe → N^bik^) LMCT (NCS → Fe) LL'CT (NCS → N^bik^) LL'CT (NCS → N^bik^) d-d (Fe → Fe)
Low-spin[Fe^II^(^Me^bik)_2_(NCS)_2_]	329 354 448 506 611	30383 28273 22301 19765 16377	0.081 0.026 0.011 0.005 0.045	π -π^*^ (N^bik^ → N^bik^) MLCT (Fe → N^bik^) LL'CT (NCS → N^bik^) LL'CT (NCS → N^bik^) MLCT (Fe → N^bik^)
Fe^II^(^Me^bik)_2_(NCSe)_2_]	351 429 478 596	28457 23303 20931 16769	0.023 0.005 0.015 0.074	LL'CT (NCS → N^bik^) MLCT (Fe → N^bik^) LL'CT (NCS → N^bik^) MLCT (Fe → N^bik^)

a *MLCT, LMCT and LL'CT denoting transitions with a main character of metal-to-ligand, ligand-to-metal and ligand-to-ligand charge transfer*.

## Conclusion

The current report presents a straightforward synthesis of two new air-stable Fe(II) spin-crossover complexes based on β-diimine ligands, namely [Fe(^Me^bik)_2_(NCS)_2_], **1**, and [Fe(^Me^bik)_2_(NCSe)_2_], **2**, which are reminiscent of the well-known [Fe^II^(phen)_2_(NCX)_2_] complexes. The magnetic studies reveal the occurrence of weakly cooperative spin transition. This is coherent with the structural analyses, which show only weak intermolecular interactions (pseudo-hydrogen bonds) between the SCO complexes. The transitions are perfectly reversible upon heating and cooling, which is in agreement with the absence of volatile solvent molecule in the crystal lattices of both compounds. Actually, both compounds do not significantly lose their crystallinity upon heating. This has allowed the structural determination of the LS and HS states in both cases, revealing the lengthening of the metal-ligand distances and the higher distortion of the coordination sphere in the HS state.

The spin transition are centered at 240 and 330 K, in **1** and **2** respectively, in agreement with the higher ligand field induced by the NCSe ligand compared to the NCS one. The theoretical calculations performed on the electronic structures of both low-spin and high-spin structures of **1** and **2** support the occurrence of a spin-transition. They also enlighten the bonding frame in the two complexes: the M-NCSe bond is more covalent than the M-NCS one. In addition, the back-donation from the Fe to the ^Me^bik ligand is also more efficient in the NCSe complex and it leads to a stronger ligand field. Both effects favor a higher spin-transition temperature in the NCSe derivative, as previously observed in [Fe^II^(phen)_2_(NCX)_2_] derivatives. Several functionals and variation of % HF exchange were explored to accurately reproduce the HS-LS gap for these two complexes. These attempts reveal that lower % HF exchange such as the one present in B3LYP^*^ functional lead to superior results for estimating the gap while it worsens the estimation of the thermodynamic parameters. In other words, a universal functional, which could accurately compute spin-state splitting, thermodynamic parameters and geometries is unfounded.

Interestingly, a LIESST effect is observed at low temperature in both complexes. However, in contrast with other related [Fe^II^(^R^bik)_2_(NC-)_2_]^2+^ SCO complexes, the relaxation temperature are very low here, and the relaxation of the magnetization occurs when the light is switched off. The experiments also reveal a remarkable dependence of the photomagnetic effect on the irradiation wavelengths. In the present case, a LIESST effect is observed when irradiating the sample in the MLCT band (near 600 nm) but with a weak efficiency. The most efficient photo-switching of the magnetization occurs at 900 nm, in the near IR.

Finally, it is worth noting that the comparison of these results with previous photomagnetic measurements on related [Fe(^R^bik)_2_(NC-)_2_] complexes show that the most efficient wavelength can be varied by changing the nature of the –N donor ligands or metallo-ligands. The rationalizing of this dependency is difficult. At that stage, advanced physical measurements would be useful, for example, to identify the nature of the excited states involved in the LIESST effect. This might be a necessary would be of very high value to gain control on the photomagnetic effect by rational chemical design.

## Materials and methods

### Synthesis

The ^Me^bik ligand was synthesized according to a literature procedure (Lucas et al., 2000). Commercially available chemicals including KSCN, KSeCN, and Fe(SO_4_)^.^7H_2_O were purchased from commercial sources and used without prior purification.

### Preparation of [Fe(^Me^bik)_2_(NCS)_2_] (1) and [Fe(^Me^bik)_2_(NCSe)_2_] (2)

A solution of ^Me^bik (2.5 mmol) in water (5 mL) was added to a solution of Fe(SO_4_).7H_2_O (1.2 mmol) in the same solvent (30 mL). The resulting deep blue solution was stirred for few minutes. Then a freshly prepared solution of KSCN/ KSeCN (16 mmol) in water (5 mL) was quickly added to the blue reaction mixture. A green precipitate immediately appeared. The turbid mixture was allowed to stir for 30 min. The precipitate was isolated by water suction, washed vigorously with distilled water and dried in vacuum. The crude product was dissolved in a minimum amount of DMSO at 60°C and the resulting bluish gray solution (*ca*. 10 mL) was stirred for 30 min and filtered. Slow evaporation of the filtrate under ambient condition afforded in 2 weeks dark blue plate like crystals of **1** and **2** that were suitable for single-crystal X-ray diffraction. Yields: 81% (**1**), 86% (**2**). IR (ATR): **(1)** ν_max_/cm^−1^: 3147, 3127, 2959, 2100 (NC_NCS_), 2066 (NC_NCS_), 1639 (CO_bik_), 1520, 1484, 1417, 1287, 1169, 897 cm^−1^. (**2**):: ν_max_/cm^−1^: 3144, 3112, 2953, 2097 (NC_NCSe_), 2069 (NC_NCSe_), 1628 (CO_bik_), 1518, 1482, 1416, 1288, 1170, 892 cm^−1^. Anal. Calcd for C_20_H_20_FeN_10_O_2_Se_2_: C, 37.17; H, 3.12; N, 21.68 Found: C, 37.06; H, 3.27 N, 21.31. Anal. Calcd for C_20_H_20_FeN_10_O_2_S_2_: C, 43.48; H, 3.65; N, 25.36 Found: C, 43.10; H, 3.70; N, 25.01.

### X-ray data collection and structure refinement

A single crystal of **1** and **2** compound were selected, mounted onto a cryoloop, and transferred in a cold nitrogen gas stream. Intensity data were collected with a BRUKER Kappa-APEXII diffractometer with graphite-monochromated Mo-Kα radiation (λ = 0.71073 Å). Data collection were performed with APEX2 suite (BRUKER). Unit-cell parameters refinement, integration, and data reduction were carried out with SAINT program (BRUKER). SADABS (BRUKER) was used for scaling and multi-scan absorption corrections. In the WinGX suite of programs (Farrugia, 1999), the structure were solved with SHELXT-14 (Palatinus and Chapuis, 2007) program and refined by full-matrix least-squares methods using SHELXL-14 (Sheldrick, 2015). The Bilbao Crystallograhic Server (de la Flor et al., 2016) was used to compare the crystal structure of **1** measured at 200 K to others because it was recorded in a different setting.

CCDC 1844949-1844955 contain the supplementary crystallographic data for this paper. These data can be obtained free of charge from The Cambridge Crystallographic Data Centre via www.ccdc.cam.ac.uk/data_request/cif.

### Analytical and spectroscopic measurements

***Elemental analyses*** for C, H, and N were performed on a Perkin-Elmer 240C analyser at the ISCN (Gif sur Yvette, France).

***FTIR spectroscopic data*** were carried out on a Vertex 70 Bruker instrument working in the ATR mode and collected in the 400–4000 cm^−1^ range at room temperature (with a 4 cm^−1^ resolution).

***Solid-state UV-vis spectra*** were measured in the range of 350–1200 nm on a CARY 5000 double-beam spectrophotometer equipped with the Eurolabo variable-temperature cell (21525, quartz windows) and Specac temperature controller. The measurements were performed on KBr pellets. ~2 mg of fresh crystallites of compounds were dispersed without any grinding in *ca*. 99 mg of KBr, this latter being previously ground. This sample preparation aimed at minimizing the formation of crystalline defects that could alter the SCO characteristics.

### Magnetic measurements

Magnetic susceptibility data were collected using a Quantum Design SQUID magnetometer (MPMS-5S Model) calibrated against a standard palladium sample. The magnetic susceptibility values were corrected from the diamagnetism of the molecular constituents and of the sample holder. The measurement was carried out on fresh crystals of **1** and **2** in the temperature range 4–400 K. Magnetic field of 1 T was used for the study.

Photomagnetic measurements were carried out by using a sample holder equipped with an optical fiber. In a typical experiment 0.5 mg of finely ground crystals were deposited on an adhesive tape. The sample was separated from the end of the fiber by 5.5 cm. All the irradiations were carried out at 20 K to minimize the temperature variation induced by light. Both the samples were irradiated with laser diodes of wavelength 405, 532, 635, 808, 900, and 1313 nm (ca. 5–10 mW cm^−2^). The experimental data were corrected in a similar way to that of bulk magnetic susceptibility measurement.

### Theoretical details

DFT calculations were performed on the X-ray structural coordinates using the Gaussian 09 (Frisch et al., 2009) suite programs for complexes **1**-**2** in 200, 300, and 400 K structures. In order to investigate the dependence of the spin state energetic on the amount of exact exchange in the B3LYP functional, the %HF exchange is varied systematically from 0 to 25% also by adjusting the DFT exchange accordingly (Becke, 1988) and geometry optimization followed by the frequency calculation is performed in the DMSO solvent environment using PCM solvent model (Tomasi et al., 2005). Additional frequency calculations are also performed on top of the optimized coordinates by incorporating the dispersion correction (D2) recommended by Grimme (2006) Additional frequency calculations were performed to extract the dispersion corrected thermodynamic parameters. All calculations employ Ahlrichs polarized triple-ζ valence (TZVP) basis set for iron, nitrogen, sulfur, selenium and single-ζ valence (SVP) basis set for rest of the atoms (Schäfer et al., 1992, 1994). The geometry optimization and frequency calculations were also performed using OPBE(Perdew et al., 1997; Handy and Cohen, 2001) functional as this is shown to be superior in estimating thermodynamic quantities (Kepp, 2013). Beside, the time dependent DFT (TDDFT) calculations were performed on the low spin and high spin structures of complexes **1** and **2** using ORCA 3.0.3 suite (Neese, 2012) by employing B3LYP (Becke, 1988) as functional along with def2-TZVP (Schäfer et al., 1992, 1994; Weigend and Ahlrichs, 2005) basis set for iron, sulfur and selenium, and TZVP (Schäfer et al., 1992, 1994; Weigend and Ahlrichs, 2005) basis set for the rest of the atoms also by incorporating the solvent effects using COSMO (Tomasi et al., 2005; Ye and Neese, 2010b) method. TDDFT calculations were performed in order to understand the absorption properties of these complexes.

## Author contributions

SD and HS prepared the samples and made the measurments. AF provided some help in the NMR measurements. YL has made some of the magnetic measurements. L-MC carried out the XRD analyses. RL supervised the work and wrote the draft. ST carried out the theoretical calculation and participate to the writing process. GR supervised the theoretical study and contributed to writing the article. M-LB supervised part of the work and the UV-vis measurement, and actively contributed to the improvement of the article.

### Conflict of interest statement

The authors declare that the research was conducted in the absence of any commercial or financial relationships that could be construed as a potential conflict of interest.
