# Supplementary material for: Thermally-Induced Spin Crossover and LIESST Effect in the Neutral [FeII(Mebik)2(NCX)2] Complexes: Variable-Temperature Structural, Magnetic, and Optical Studies (X = S, Se; Mebik = bis(1-methylimidazol-2-yl)ketone)
Source: Front Chem. 2018 Aug 21;6:326. doi: 10.3389/fchem.2018.00326 (PMC6111792; doi:10.3389/fchem.2018.00326)
Supplement: Supplementary file 1 [file Table_1.DOCX]

Supplementary Material

# Thermally-induced spin crossover and LIESST effect in the neutral [Fe^II^(^Me^bik)_2_(NCX)_2_] complexes:

# variable-temperature structural, magnetic and optical studies

(X = S, Se ; ^Me^bik = bis(1-methylimidazol-2-yl)ketone)

**Siddhartha De, Lise-Marie Chamoreau, Hasnaa El Said, Yanling Li, Marie-Laure Boillot, Subrata Tewary, Gopalan Rajamaran, Alexandrine Flambard, and Rodrigue Lescouëzec^*^**

*** Correspondence:** Rodrigue.lescouezec@sorbonne-universite.fr

**Table S1 | Crystallographic data for 1 at 200, 300 and 400 K.**

| Formula, M_w_ [g mol^-1^]  (crystal system) | (**1**) C_20_ H_20_ Fe N_10_ O_2_ S_2_ , 552.43 g/mol  monoclinic (C2/c) | | |
| --- | --- | --- | --- |
| Temperature (K) | 200(2) | 300(2) | 400(2) |
| Density (Mg/m^3^) | 1.556 | 1.504 | 1.476 |
| Absorption coef. (mm^-1^) | 0.858 | 0.829 | 0.814 |
| F(000) | 1136 | 1136 | 1136 |
| θ range for data collection | 2.953 to 30.056° | 2.822 to 25.366° | 2.807 to 26.062° |
| Reflections collected | 13331 | 12305 | 12846 |
| Independent reflections | 3439 | 2239 | 2453 |
| R(int) | 0.0315 | 0.0343 | 0.0239 |
| data / restraints / parameters | 3439 / 0 / 161 | 2239 / 0 / 161 | 2453 / 0 / 161 |
| Goodness-of-fit on F2 | 1.030 | 0.998 | 1.023 |
| Final R indices | R1 = 0.0394 | R1 = 0.0387 | R1 = 0.0370 |
| [*I* > 2σ(*I*)] | wR2 = 0.0870 | wR2 = 0.0805 | wR2 = 0.0887 |
| R indices (all data) | R1 = 0.0590 | R1 = 0.0635 | R1 = 0.0577 |
|  | wR2 = 0.0937 | wR2 = 0.0890 | wR2 = 0.0979 |
| Largest diff. peak  and hole [e.Å-3] | 0.408 and -0.262 | 0.278 and -0.222 | 0.357 and -0.282 |
| CCDC number | 1844949 | 1844950 | 1844951 |

wR2 = {Σ [w(Fo^2^–Fc^2^)^2^] / Σ[w(Fo^2^)^2^]}^1/2^

R1 = Σ| |Fo| – |Fc| | / Σ|Fo|

**Table S2 | Crystallographic data for 2 at 200, 300 and 400 K.**

| Formula, M_w_ [g mol^-1^]  (crystal system) | (**2**) C_20_ H_20_ Fe N_10_ O_2_ Se_2_, 6 46.23 g/mol  monoclinic (C2/c) | | |
| --- | --- | --- | --- |
| Temperature (K) | 200(2) | 300(2) | 400(2) |
| Density (Mg/m^3^) | 1.761 mg/m3 | 1.732 mg/m3 | 1.665 mg/m3 |
| Absorption coef. (mm^-1^) | 8.723 mm-1 | 8.578 mm-1 | 8.247 mm-1 |
| F(000) | 1280 | 1280 | 1280 |
| θ range for data collection | 3.974 to 70.090° | 6.324 to 70.301° | 3.905 to 68.414° |
| Reflections collected | 10448 | 6825 | 14464 |
| Independent reflections  R(int) | 2254  0.0178 | 2296  0.0306 | 2367  0.0338 |
| Data / restraints/parameters | 2254 / 0 / 161 | 2296 / 0 / 161 | 2367 / 0 / 161 |
| Goodness-of-fit on F2 | 1.068 | 1.044 | 1.066 |
| Final R indices  [*I* > 2σ(*I*)] | R1 = 0.0218 | R1 = 0.0392 | R1 = 0.0382 |
|  | wR2 = 0.0536 | wR2 = 0.1013 | wR2 = 0.0949 |
| R indices (all data) | R1 = 0.0223 | R1 = 0.0415 | R1 = 0.0476 |
|  | wR2 = 0.0537 | wR2 = 0.1037 | wR2 = 0.1003 |
| Largest diff. peak  and hole [e.Å-3] | 0.372 and -0.298 | 0.966 and -0.642 | 0.740 and -0.664 |
| CCDC number | 1844952 | 1844953 | 1844955 |

wR2 = {Σ [w(Fo^2^–Fc^2^)^2^] / Σ[w(Fo^2^)^2^]}^1/2^

R1 = Σ| |Fo| – |Fc| | / Σ|Fo|

CCDC 1844954 contains crystallographic data collected at 360K, which are not commeneted in this article

**Table S3 | Selected inter-atomic distances (Å) and angles (deg.) of the coordination sphere and the NCS ligand in the complex [Fe(^Me^bik)_2_(NCS)_2_] (1) at 200, 300, and 400 K.**

|  | **200** | **300** | **400** |
| --- | --- | --- | --- |
| Fe(1)-N(1) _NCS_ | 1.967(16) | 2.108(3) | 2.133(2) |
| Fe(1)-N(2) _bik_ | 1.970(15) | 2.140(2) | 2.164(2) |
| Fe(1)-N(4) _bik_ | 1.965(14) | 2.142(2) | 2.174(18) |
| N(1a)-Fe(1)-N(1) | 93.84(9) | 94.83(14) | 95.10(13) |
| N(1a)-Fe(1)-N(2) | 87.09(6) | 89.77(9) | 89.95(9) |
| N(1)-Fe(1)-N(2) | 92.50(6) | 93.28(9) | 93.58(8) |
| N(1a)-Fe(1)-N(2a) | 92.50(6) | 93.28(9) | 93.58(9) |
| N(1)-Fe(1)-N(2a) | 87.09(6) | 89.77(9) | 89.95(9) |
| N(2)-Fe(1)-N(2a) | 179.40(9) | 175.49(12) | 174.76(11) |
| N(1a)-Fe(1)-N(4) | 177.08(6) | 174.64(9) | 174.08(8) |
| N(1)-Fe(1)-N(4) | 88.75(6) | 89.81(9) | 89.79(8) |
| N(2)-Fe(1)-N(4a) | 91.45(6) | 92.66(8) | 93.06(7) |
| N(2a)-Fe(1)-N(4a) | 88.98(6) | 84.03(8) | 83.08(7) |
| N(1a)-Fe(1)-N(4a) | 88.75(6) | 89.81(9) | 89.80(8) |
| N(1)-Fe(1)-N(4a) | 177.08(6) | 174.64(9) | 174.08(8) |
| N(2)-Fe(1)-N(4) | 88.98(6) | 84.03(8) | 83.08(7) |
| N(2a)-Fe(1)-N(4) | 91.45(6) | 92.66(8) | 93.06(7) |
| N(4a)-Fe(1)-N(4) | 88.69(8) | 85.69(12) | 85.52(11) |
| S(1)-C(1) | 1.639(19) | 1.624(3) | 1.616(3) |
| N(1)-C(1) | 1.163(2) | 1.154(3) | 1.147(3) |
| C(1)-N(1)-Fe(1) | 164.90(15) | 159.40(2) | 158.60(2) |
| N(1)-C(1)-S(1) | 178.77(16) | 178.80(3) | 178.90(2) |

Atoms with and without the label “a” are related by the two fold axis along the c axis through the iron atom of the molecule

**Table S4 | Selected inter-atomic distances (Å) and angles (deg.) of the coordination sphere and the NCSe ligand in the complex [Fe(^Me^bik)_2_(NCSe)_2_] (1) at 200, 300, and 400 K.**

|  | **200** | **300** | **400** |
| --- | --- | --- | --- |
| Fe(1)-N(1) _NCSe_ | 1.961 (16) | 1.973(3) | 2.132(3) |
| Fe(1)-N(2) _bik_ | 1.959 (16) | 1.988(2) | 2.144(3) |
| Fe(1)-N(4) _bik_ | 1.962 (17) | 1.991(3) | 2.137(3) |
| N(2a)-Fe(1)-N(2) | 89.32(9) | 88.96(14) | 87.62(16) |
| N(2a)-Fe(1)-N(1a) | 88.66(7) | 88.75(11) | 89.57(13) |
| N(2)-Fe(1)-N(1a) | 177.52(7) | 177.60(10) | 175.67(13) |
| N(2a)-Fe(1)-N(1) | 177.52(7) | 177.60(10) | 175.67(13) |
| N(2)-Fe(1)-N(1) | 88.66(7) | 88.75(11) | 89.57(13) |
| N(1a)-Fe(1)-N(1) | 93.40(10) | 93.55(16) | 93.4(2) |
| N(2a)-Fe(1)-N(4) | 91.76(7) | 92.16(10) | 93.79(11) |
| N(2)-Fe(1)-N(4) | 89.10(6) | 88.11(10) | 83.74(11) |
| N(1a)-Fe(1)-N(4) | 92.40(7) | 92.71(11) | 93.17(13) |
| N(1)-Fe(1)-N(4) | 86.76(7) | 87.04(11) | 89.17(14) |
| N(2a)-Fe(1)-N(4a) | 89.11(6) | 88.11(10) | 83.74(11) |
| N(2)-Fe(1)-N(4a) | 91.76(7) | 92.16(10) | 93.79(11) |
| N(1a)-Fe(1)-N(4a) | 86.76(7) | 87.04(11) | 89.17(14) |
| N(1)-Fe(1)-N(4a) | 92.40(7) | 92.70(11) | 93.17(13) |
| N(4)-Fe(1)-N(4a) | 178.78(9) | 179.62(15) | 176.59(16) |
| Se(1)-C(1) | 1.797(2) | 1.785(3) | 1.770(4) |
| N(1)-C(1) | 1.154(3) | 1.165(4) | 1.137(5) |
| C(1)-N(1)-Fe(1) | 165.42(16) | 165.10(3) | 159.20(4) |
| N(1)-C(1)-Se(1) | 178.68(17) | 178.50(3) | 178.60(4) |

Atoms with and without the label “a” are related by the two fold axis along the c axis through the iron atom of the molecule

**Table S5 | Intermolecular distances (Å) shorter than the van de Waals and shortest Fe•••Fe distances involved in the same interactions**

| **[Fe(^Me^bik)_2_(NCS)_2_]** | | | |
| --- | --- | --- | --- |
|  | **200 K** | **300 K** | **400 K** |
| C(3)-S(1) | 3.652 | 3.682 | 3.708 |
| C(3)-C(1) | 3.599 | 3.574 | 3.606 |
| C(4)-S(1) | 3.797 | 3.783 | 3.816 |
| N(3)-S(1) | 3.782 | 3.832 | 3.861 |
| Fe•••Fe | 7.774 | 7.648 | 7.681 |
| **[Fe(^Me^bik)_2_(NCSe)_2_]** | | | |
|  | **200 K** | **300 K** | **400 K** |
| C(7)-Se(1) | 3.772 | 3.795 | 3.801 |
| C(7)-C(1) | 3.681 | 3.693 | 3.696 |
| C(10)-Se(1) | 3.828 | 3.853 | 3.861 |
| N(5)-Se(1) | 3.809 | 3.850 | 3.897 |
| Fe•••Fe | 7.916 | 7.918 | 7.849 |

**Supplementary Figure S1 | View of the pseudo H-bond interaction (dotted green lines) in 2 at 200 K (view along *c* axis).**

**Supplementary Figure S2 | Temperature dependence of the UV-vis solid-state spectra of 2 between 125 and 375 K .**

| **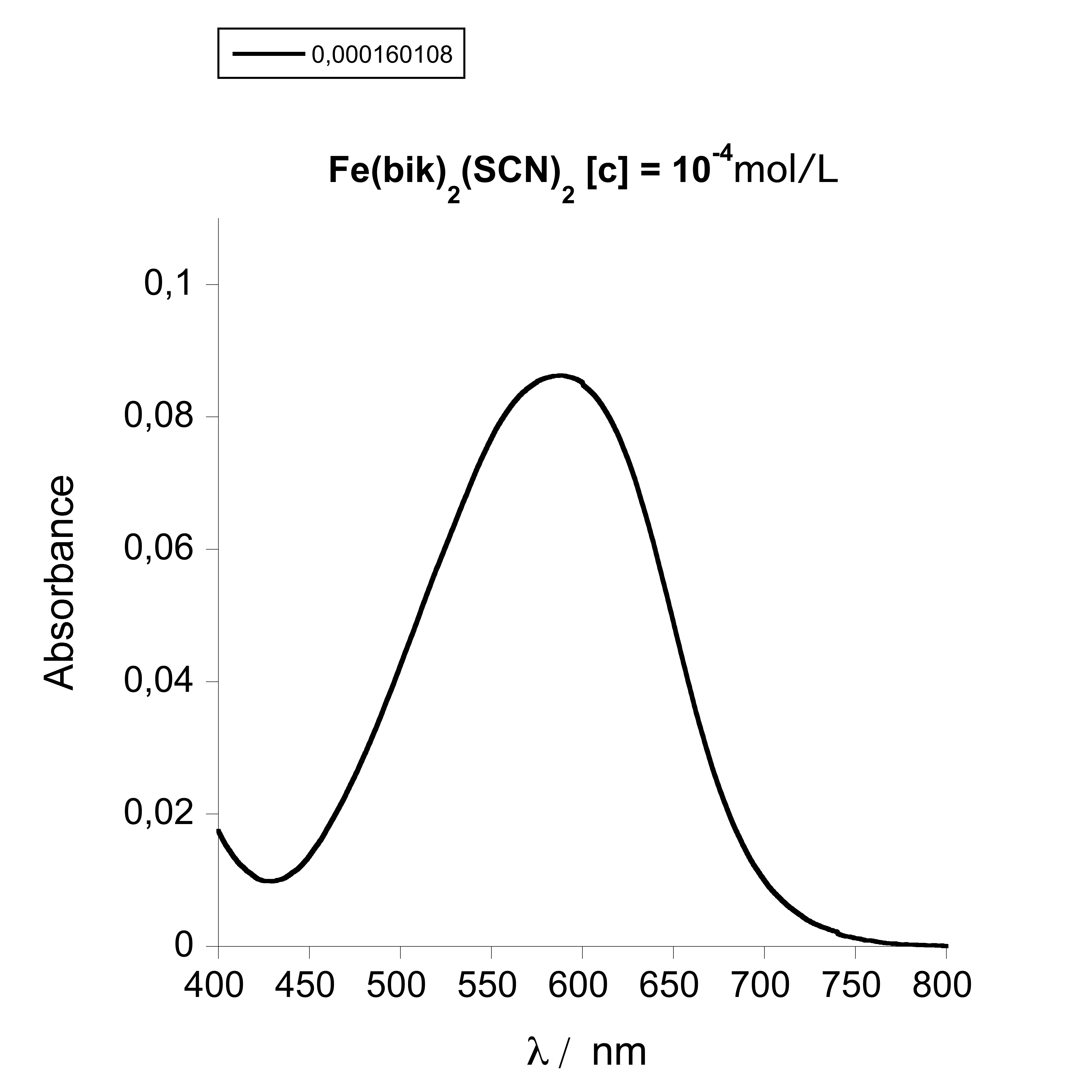** | **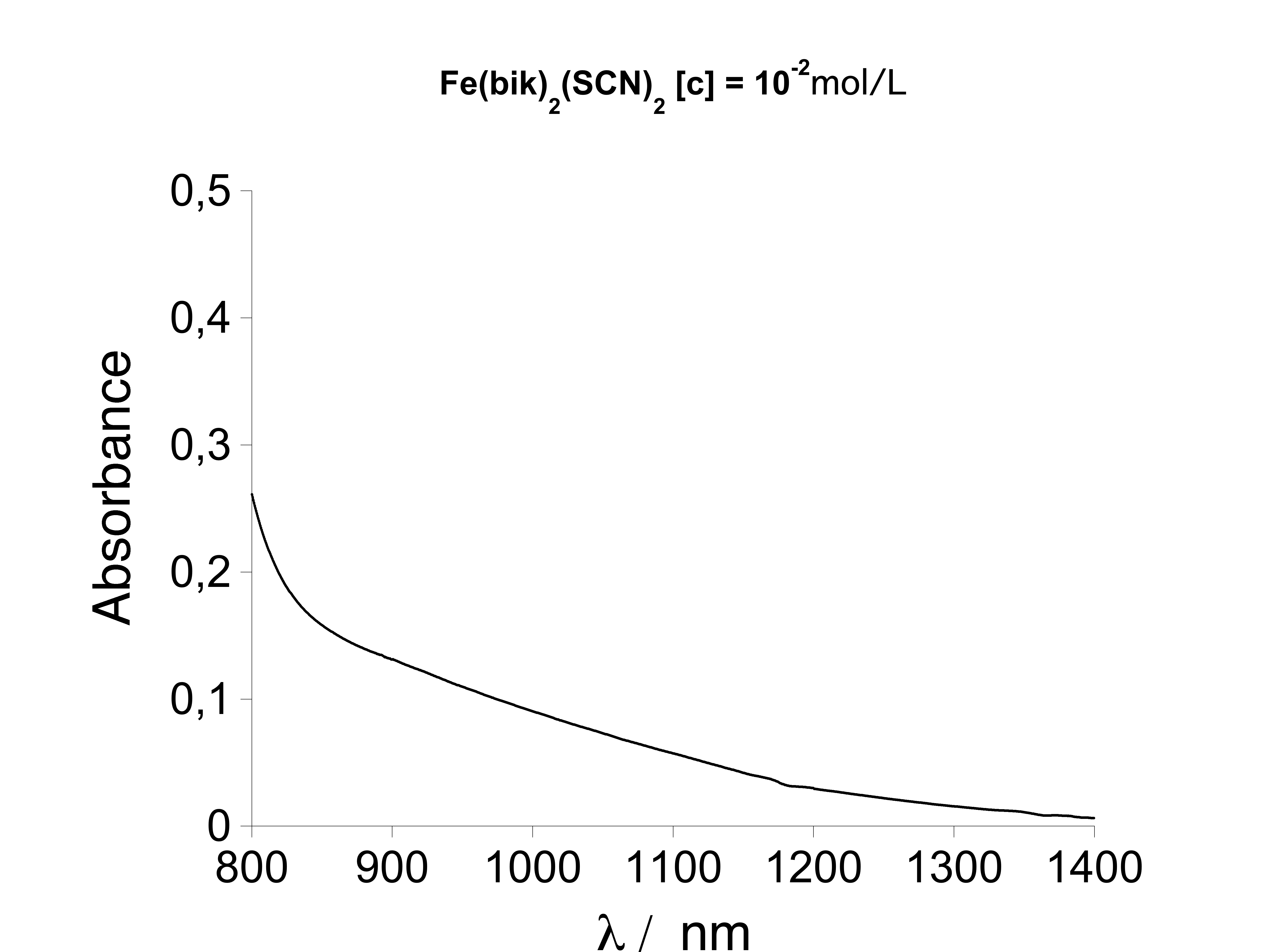** |
| --- | --- |
| **1** (10^-4^ mol/L) | **1** (10^-2^ mol/L) |
| **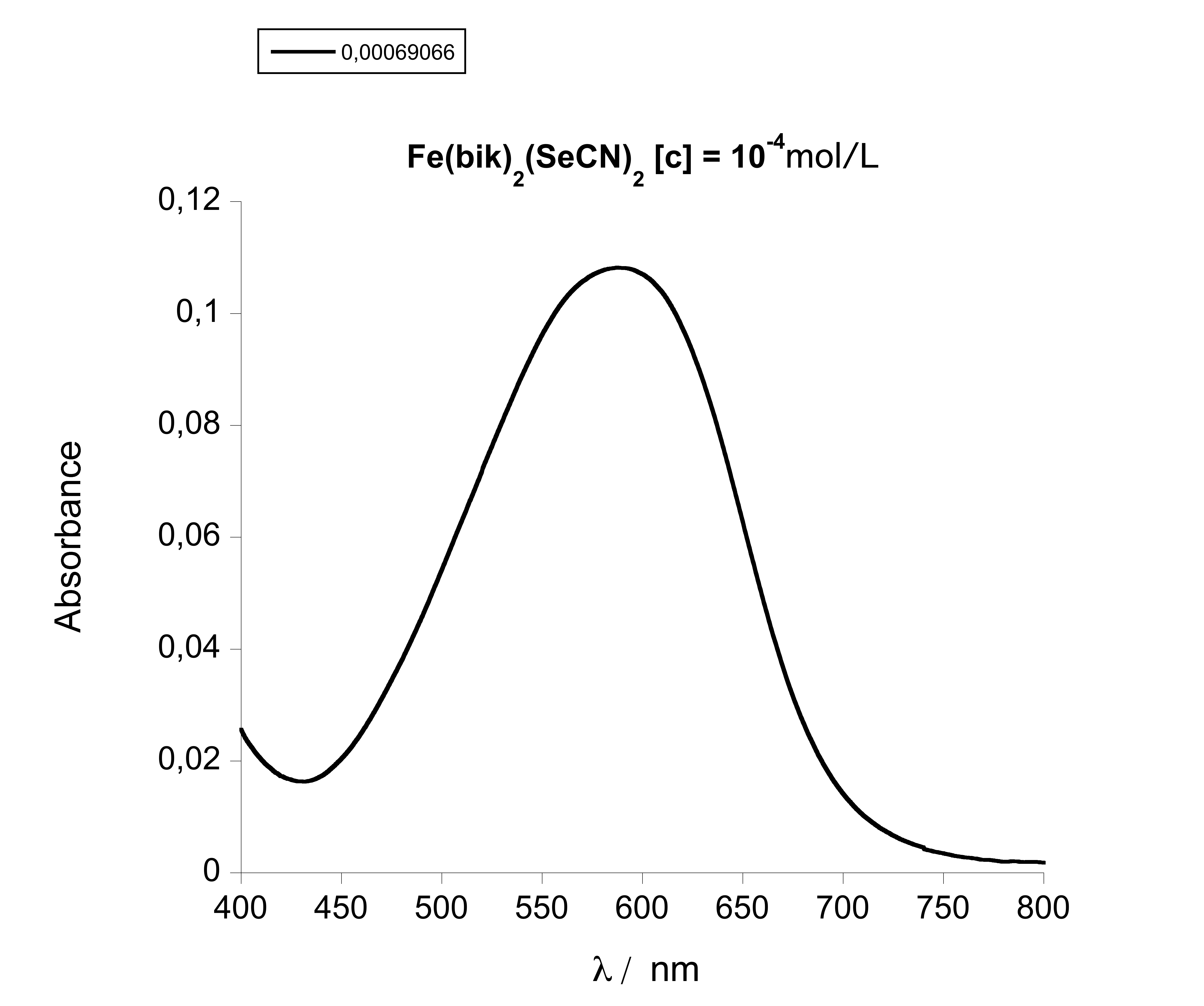** | **** |
| **2** (10^-4^ mol/L) | **2** (10^-2^ mol/L) |

**Supplementary Figure S3 | UV-vis solution spectra (Absorbance versus wavelength) of 1 and 2 in DMSO at room temperature.**

**Theoretical part**

**Supplementary Table S6 | Some selected bond parameters from B3LYP* and OPBE computed geometries for 1 and 2 in both high spin and low spin geometries.**

| **B3LYP*** | [Fe(bik)_2_(NCS)_2_] (**1**) | | [Fe(bik)_2_(NCSe)_2_] (**2**) | |
| --- | --- | --- | --- | --- |
| Spin state | LS | HS | LS | HS |
| *d*[Fe-N(L)]_av_ | 2.006 | 2.229 | 2.005 | 2.229 |
| *d*[(Fe-NCX)]_av_ | 1.969 | 2.126 | 1.964 | 2.129 |
| axial (N-Fe-N)_av_ angle | 179.4 | 172.8 | 179.5 | 175.1 |
| bite (N-Fe-N)_av_ angle | 88.1 | 86.6 | 86.0 | 87.2 |
| [Fe-N-C(X)]_av_ angle | 178.3 | 174.2 | 176.9 | 175.4 |

| **OPBE** | [Fe(bik)_2_(NCS)_2_] (**1**) | | [Fe(bik)_2_(NCSe)_2_] (**2**) | |
| --- | --- | --- | --- | --- |
| Spin state | LS | HS | LS | HS |
| *d*[Fe-N(L)]_av_ | 1.948 | 2.221 | 1.950 | 2.093 |
| *d*[(Fe-NCX)]_av_ | 1.907 | 2.083 | 1.901 | 2.216 |
| axial (N-Fe-N)_av_ angle | 178.9 | 173.0 | 178.8 | 173.6 |
| bite (N-Fe-N)_av_ angle | 88.0 | 85.6 | 88.0 | 85.5 |
| [Fe-N-C(X)]_av_ angle | 176.6 | 173.6 | 175.8 | 175.2 |

**
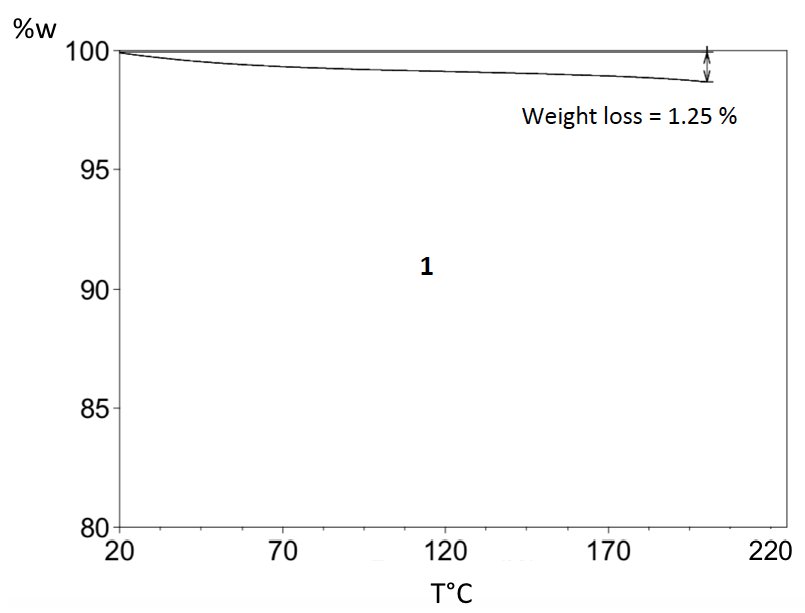
**

**
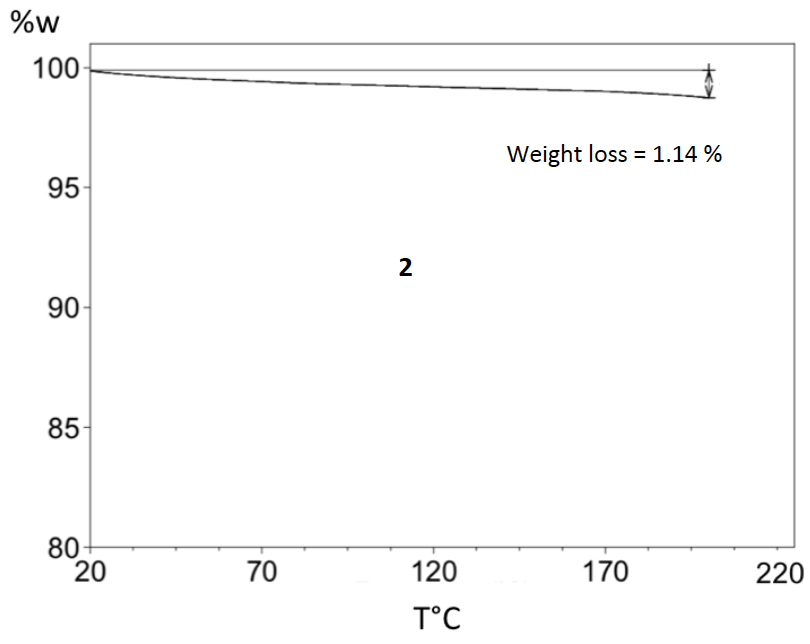
**

**Supplementary Figure S4 | Thermo-gravimetric analysis of 1 and 2 under N_2_ flux (2°C / min).**

The very small weight loss betwee room temperature and ca. 120° is ascribed to DMSO solvent molecules adsorbed at the surface of the microcrystals.

**
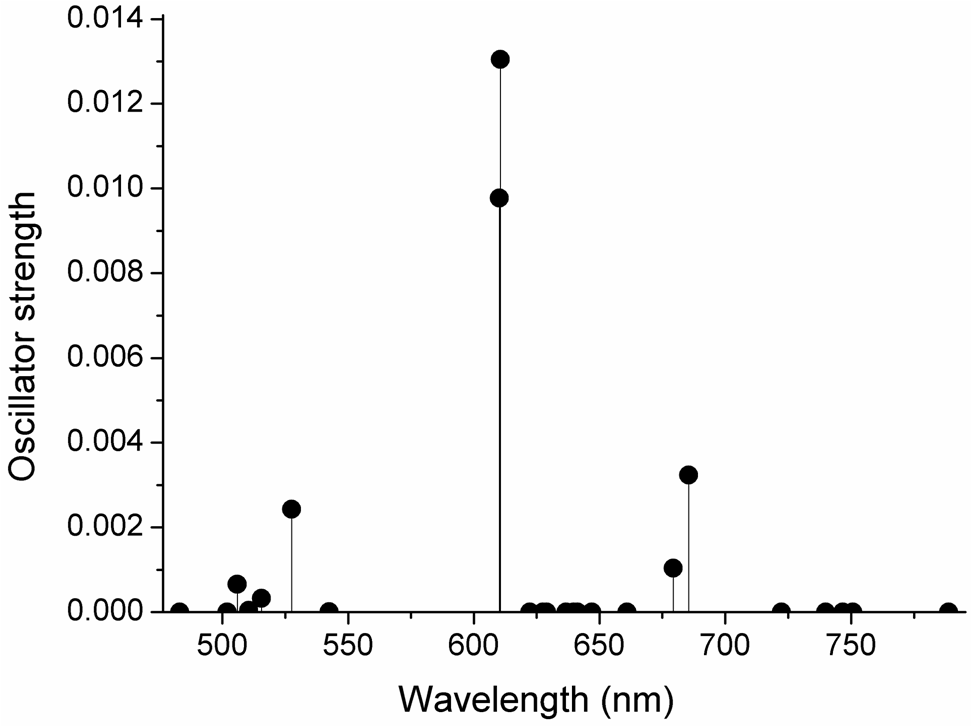
**

**Supplementary Figure S5 | TDDFT computed absorption spectra of complex 1 in the low-spin state depicting important electronic transitions.**

**
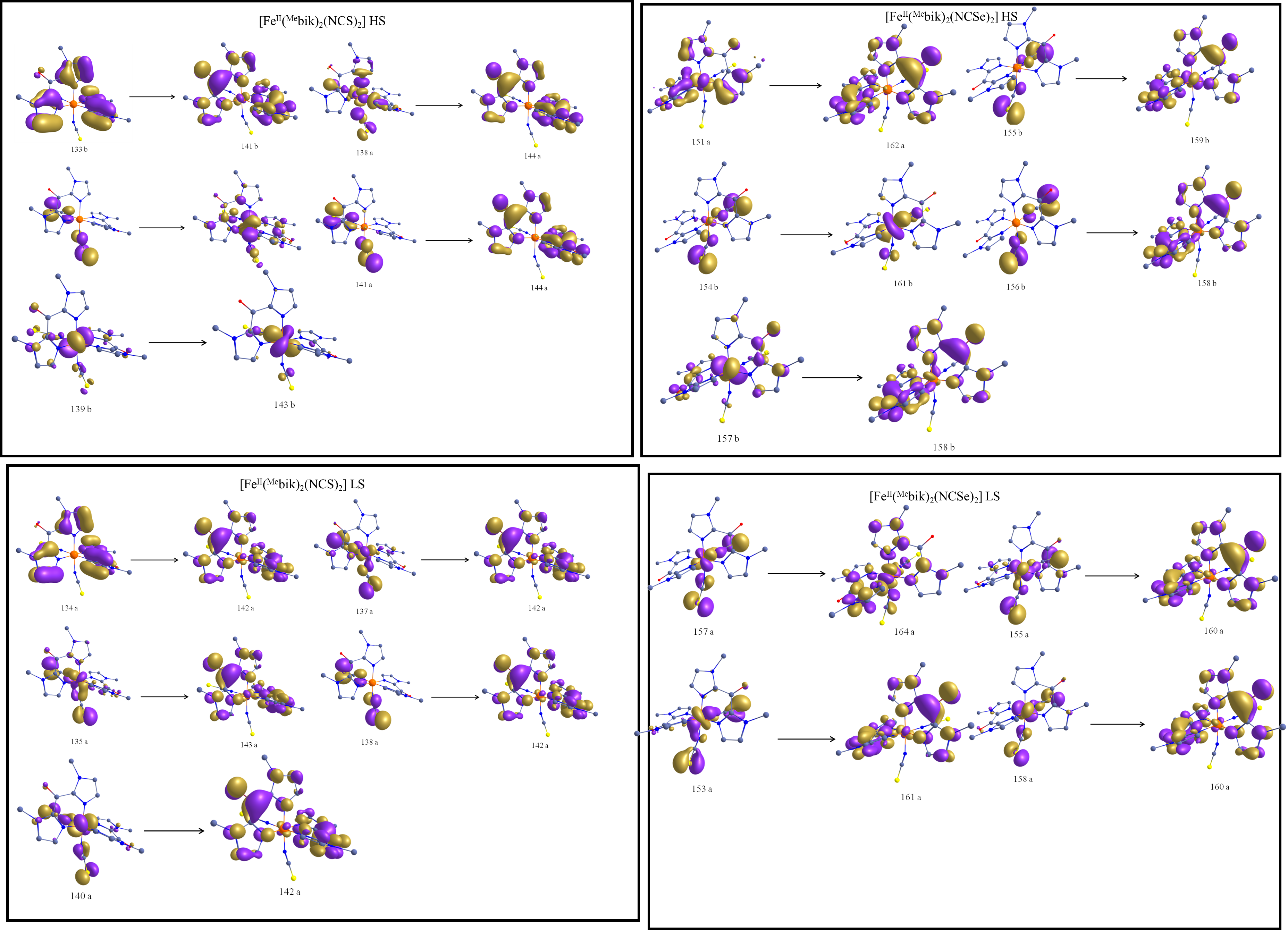
**

**Supplementary Figure S6 | Orbital diagram corresponding to transitions observed in the TDDFT computed absorption spectra of complexes 1 and 2 using HS and LS X-ray structure.**
